# Tables of the Inverse Laplace Transform of the Function $e^{-s^{\beta }}$

**DOI:** 10.6028/jres.095.036

**Published:** 1990

**Authors:** Menachem Dishon, John T. Bendler, George H. Weiss

**Affiliations:** National Institute of Standards and Technology, Gaithersburg, MD 20899; General Electric Corporate Research and Development, Schenectady, NY 12301; National Institutes of Health, Bethesda, MD 20892

**Keywords:** numerical inversion of Laplace transforms, relaxation processes, stable laws, stretched exponentials

## Abstract

The inverse transform, $g\left(t\right)=L^{-1}\left(e^{-s^{\beta }}\right)$, 0 < *β* < 1, is a stable law that arises in a number of different applications in chemical physics, polymer physics, solid-state physics, and applied mathematics. Because of its important applications, a number of investigators have suggested approximations to *g*(*t*). However, there have so far been no accurately calculated values available for checking or other purposes. We present here tables, accurate to six figures, of *g*(*t*) for a number of values of *β* between 0.25 and 0.999. In addition, since *g*(*t*), regarded as a function of *β*, is uni-modal with a peak occurring at *t* = *t*_max_ we both tabulate and graph *t*_max_ and 1/*g*(*t*_max_) as a function of *β*, as well as giving polynomial approximations to 1/*g*(*t*_max_).

## 1. Introduction

It has been known for at least 150 years that mechanical relaxation in solids is non-exponential, the decay often being characterized by a fractional power-law or logarithmic function [1,2]. It is also now generally recognized that all glassy materials exhibit non-exponential relaxation behavior both above and below the glass transition temperature, *T*_g_. This is especially clear from measurements obtained from mechanical [3–6], dielectric [7–9], and photon correlation spectroscopy [10,11]. It is also seen in measurements of volumetric [12], and thermal response [13,14].

In recent years theorists have become interested in the possibility that complex disordered systems exhibit universal features in their relaxation and transport properties, possibly arising from self-similar arrangements of obstacles to motion. This has been particularly encouraged by the observation that nearly all glassy relaxation phenomena can be described by the Kohlrausch-Williams-Watts (KWW) function

$$\phi \left(t;\tau \right)=\exp\left[-{\left(\frac{t}{\tau }\right)}^{\beta }\right],\;0<\beta <1.$$ (1)

In many physical applications it is convenient to represent *ϕ*(*t*; *τ*) in the form of a Laplace transform, which we write as

$$\phi \left(t;\tau \right)=\int_{0}^{\infty }\rho_{\tau }\left(u\right)\;e^{-\frac{t}{u}}du=\int_{0}^{\infty }e^{-vt}h_{\tau }\left(v\right)\;dv$$ (2)

where

$$h_{\tau }\left(v\right)=\frac{\rho_{\tau }\left(\frac{1}{v}\right)}{v^{2}}.$$ (3)

Thus, the function *h_τ_*(*v*) can be found as an inverse Laplace transform of the function *ϕ*(*t*;*τ*). The function *h_τ_*(*v*) has found application in the context of the theory of trap-controlled hopping in solid state physics [15,16], chromatography [17], and in the study of models for transport in disordered media [18], as well as in the deconvolution of noisy data [19].

A number of approximate algorithms have been proposed in the literature of chemical physics for the numerical evaluation of *h_τ_*(*v*) [20–25], in addition to a representation of *h_τ_*(*v*) in terms of a convergent series given by Pollard [26]. Without loss of generality we can set *τ* = 1 since *h_τ_*(*v*) can be represented in terms of the inverse transform

$$h_{\tau }\left(v\right)=\frac{\tau }{2\pi i}\int_{\Gamma }e^{v\tau s-s^{\beta }}ds$$ (4)

where Γ is a line to the right of the origin and parallel to the imaginary axis. The convergent series given by Pollard is

$$h_{1}\left(v\right)=\frac{1}{\pi }\sum_{k=1}^{\infty }{\left(-1\right)}^{k+1}\;\frac{\Gamma \left(\beta k+1\right)\sin\left(\pi \beta k\right)}{k!v^{\beta k+1}}.$$ (5)

In an earlier paper we have presented an accurate tabulation of the sine and cosine transforms of the function exp(*− t^β^*), needed for the analysis of measurements of dielectric properties taken as a function of frequency [27]. In the present paper we tabulate the inverse Laplace transform *h_τ_*(*v*). These tables may be used directly for the analysis of experimental data, but are also intended for use as a check on more easily programmed approximations, such as those suggested by earlier investigators [28–30].

## 2. Numerical Analysis

Two techniques were used to generate the tables that follow which provide an internal check on the accuracy of the computation. The first is that of numerical inversion of the Laplace transform, using a method first suggested by Dubner and Abate [31], and later given in an improved version by Crump [32]. The second is that of direct evaluation of the series given in eq (5). The approximate inverse of a Laplace transform *ĝ*(*s*) = *ℒ*{*g*(*t*)} can be expressed in the form of a Fourier series:

$$\begin{array}{c} g_{a}\left(t\right)\sim \frac{e^{at}}{T}(\frac{\hat{g}\left(a\right)}{2}+\sum_{k=1}^{\infty }[R\;e\left\{\hat{g}\left(a+i\frac{k\pi }{T}\right)\right\}\cos\left(\frac{k\pi t}{T}\right)\\ -Im\left\{\hat{g}\left(a+i\frac{k\pi }{T}\right)\right\}\sin\frac{k\pi t}{T}]) \end{array}$$ (6)

with an error, *E*(*t*) = *g_a_*(*t*) *− g*(*t*), given by

$$E\left(t\right)=\sum_{n=1}^{\infty }e^{-2naT}g\left(2nT+t\right).$$ (7)

The function $\hat{g}\left(a+i\frac{k\pi }{T}\right)$ can be written in terms of the parameters

$$b_{k}=k\pi /T,\;r_{k}=\sqrt{a^{2}+b_{k}^{2}},\;\theta_{k}=\tan^{-1}\left(b_{k}/a\right)$$

as

$$\begin{array}{l} \hat{g}\left(a+i\frac{k\pi }{T}\right)=\exp\left[-r_{k}^{\beta }\cos\left(\beta \theta_{k}\right)\right][\cos\left(r_{k}^{\beta }\sin\left(\beta \theta_{k}\right)\right)\\ \;-i\;\sin\left(r_{k}^{\beta }\sin\left(\beta \theta_{k}\right)\right)]. \end{array}$$ (8)

In eqs (6) and (7) the constants *a* and *T* are arbitrary and can be chosen to maximize accuracy in any particular application. In the present instance, in which $\hat{g}\left(s\right)=\exp\left(-s^{\beta }\right)$, the choice of these parameters is quite straightforward as will be shown below.

Equation (6) was used to evaluate the inverse transform of *ĝ*(*s*) for values of *β* in the range 0.20≤*β*≤0.999 and values of *t* ranging from 10^−8^ (for selected values of *β*) to 5, to an accuracy of at least nine significant digits. In these ranges of *β* and *t* the choice of parametric ranges *aϵ*(2.5,5) and *Tϵ*(4,8) sufficed to produce the stated accuracy. The accuracy of the numerical inversion can be checked in detail for three cases in which the inverse transforms are known exactly,

$$\beta =1/3:\;g\left(t\right)=\frac{1}{{\left(3t^{4}\right)}^{\frac{1}{3}}}Ai\left[\frac{1}{{\left(3t\right)}^{\frac{1}{3}}}\right]$$

$$\beta =1/2:\;g\left(t\right)=\frac{\exp\left(-\frac{1}{4t}\right)}{2\pi^{1/2}t^{3/2}}$$ (9)

$$\beta =2/3:\;g\left(t\right)=\frac{2^{\frac{4}{3}}\exp\left(-\frac{4}{27t^{2}}\right)}{3^{3/2}\pi^{1/2}t^{7/3}}U\left(\frac{1}{6},\frac{4}{3},\frac{4}{27t^{2}}\right)$$

where Ai(*x*) is an Airy function and *U*(*x, y, z*) is a confluent hypergeometric function [29]. Typical results for the relative error are given in table 1.

An alternative approach to the evaluation of *g*(*t*) is through the direct series shown in eq (5). The form of the series renders it useful for finding *g*(*t*) for large *t*, but the utility of the series form has occasionally been dismissed because of numerical problems associated with convergence at smaller *t.* We encountered no difficulties in finding *g*(*t*) from eq (5), provided that we used a double precision routine for the gamma functions for *k*≤22 as well as a Padé correction to Stirling’s approximation at larger *k* [33]. Thus, we write

$$\frac{\Gamma \left(1+\beta k\right)}{k!t^{\beta k+1}}\sim e^{g}\frac{P\left(\beta k\right)}{P\left(k\right)}$$ (10)

where

$$\begin{array}{l} g=k\left(1-\beta \right)+\left(\beta k-\frac{1}{2}\right)\ln(\beta k)-\left(k-\frac{1}{2}\right)\ln\left(k\right)\\ \;-\left(\beta k+1\right)\ln\left(t\right)\\ P\left(k\right)=\sum_{i=0}^{5}\frac{F_{i}}{k^{i}}. \end{array}$$ (11)

The *F_i_* are constants with the values

$$\begin{array}{l} F_{0}=1,F_{1}=\frac{1}{12},F_{2}=\frac{1}{288},F_{3}=-\frac{139}{51,840},\\ \;F_{4}=-\frac{571}{2,488,320},\text{and}\;F_{5}=\frac{163,879}{209,018,880}. \end{array}$$

## 3. Tables, Graphs, and Numerical Approximations

The inverse transform of the function *ĝ*(*s*) is tabulated in table 2 for the following values of *β*: 0.25(0.01)0.30(0.02)0.98, 0.99, 0.995, 0.997, 0.998, and 0.999. The finer intervals in *β* at low values of *β* are required because of the considerable changes in the function in that neighborhood. Spacings in *t* vary with *β* and *t* in such a way that the peaks of *g*(*t*) are most densely covered. There is little need to tabulate *g*(*t*) for *t* > 5 because for these values, the sum of no more than 10 terms of the series in eq (5) suffice to produce *g*(*t*) to six-digit accuracy for values of *β* in the interval (0.05,0.999). For example, if *β*=0.6 the sum of seven terms of the series gives *g*(10) to six places, and the sum of four terms gives *g*(100) to the same accuracy. Figures 1a–c contain graphs of *g*(*t*) as a function of *t* over the entire range of tabulated values of *β.* Note that for *β* = 1 *g*(*t*) = *d*(*t* − 1), a Dirac delta function which is represented as a vertical line in figure 1c.

**Figure 3:**
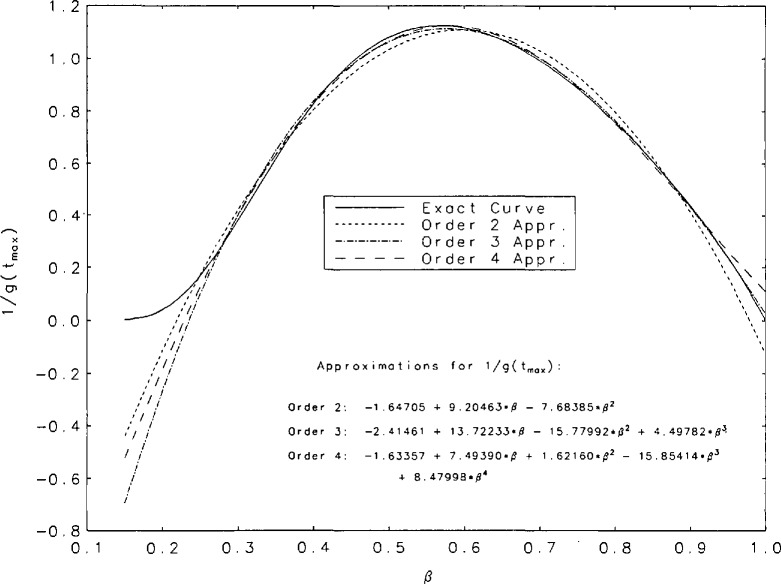
Second through fourth degree polynomial approximations to 1/*g*(*t*_max_) as a function of *β* compared to the more accurately calculated value of this quantity.

It is evident, from the curves shown in figure 1, that the *g*(*t*) are unimodal. The position of the peak will be denoted by *t*_max_. Table 3 contains some values of *t*_max_ and g(*t*_max_) for the values of *β* for which we performed our tabulations. It is interesting to observe that among the values of g(*t*_max_) there is a minimum value within the interval (0,1). Figure 2a shows graphs of *t*_max_ and 1/g(*t*_max_) as functions of *β* for values of *β* between 0.15 and 1. The minimum of g(*t*_max_) occurs at *t*_max_=0.252+ and is equal to 0.888+. These values correspond to *β*=0.567+. Figure 2b contains a plot of 1/*g*(*t*_max_) as a function of *t*_max_. Finally, we have derived polynomial least-square approximations to 1/*g*(*t*_max_) as a function of *β.* The coefficients of the approximating polynomials as well as a graphical indication of the degree of agreement with our more accurately calculated values of this function are shown in figure 3. A good approximation to 1/*g*(*t*_max_) probably requires fitting some function other than a polynomial.

## Figures and Tables

**Figure 1a:**
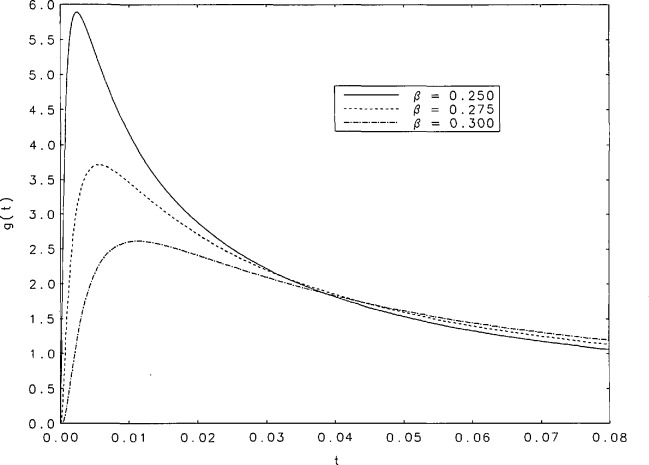
Curves of *g*(*t*) as a function of *t* in the neighborhood of the peak values for *β*=0.25, 0.275, and 0.30.

**Figure 1b:**
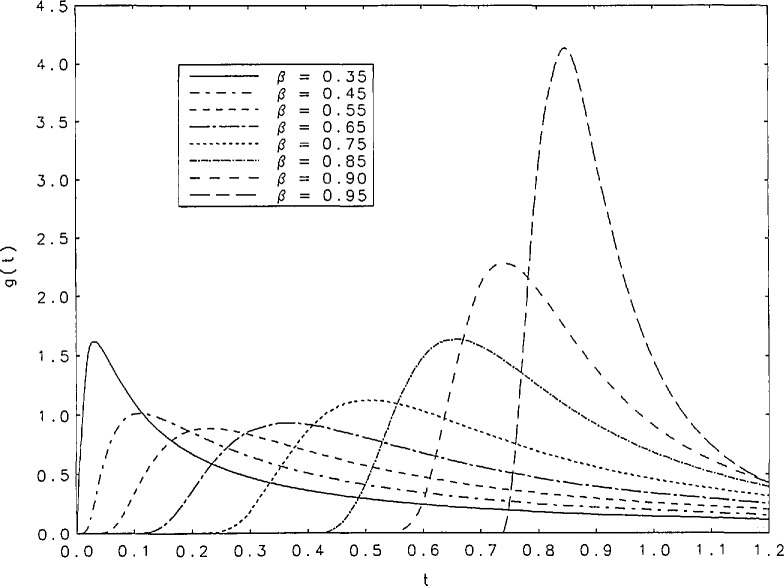
Curves of *g*(*t*) as a function of *t* in the neighborhood of the peak values for *β*=0.35(0.1)0.95.

**Figure 1c:**
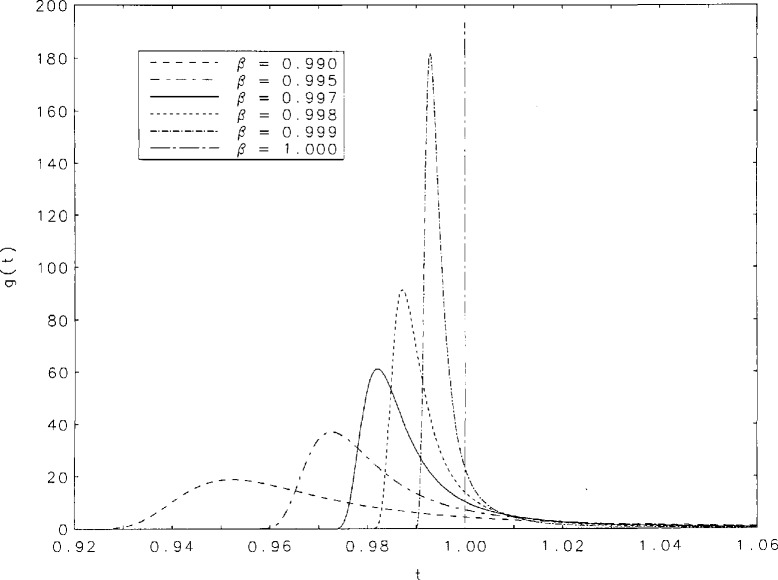
Curves of *g*(*t*) as a function of fin the neighborhood of the peak values for *β*=0.99, 0.995, 0.997, 0.998, and 0.999. The delta function at *β*=1 is indicated by the vertical line.

**Figure 2a:**
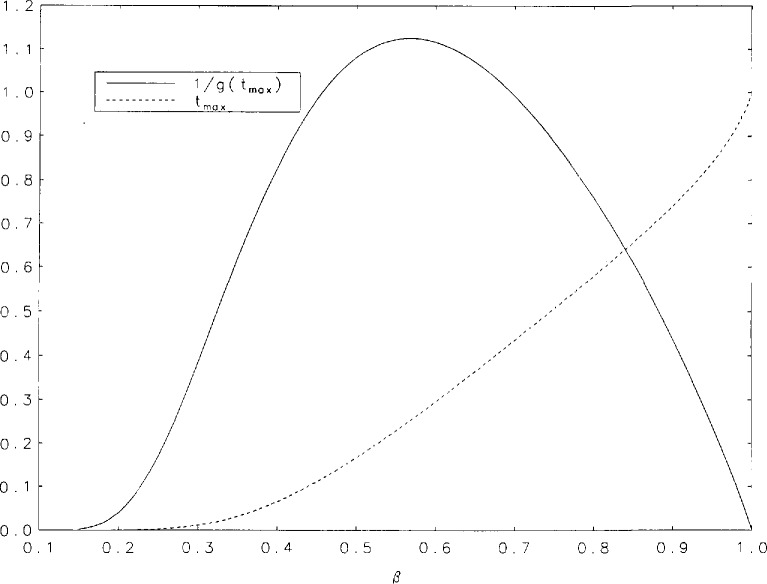
Curves of *t*_max_ and 1/*g*(*t*_max_) plotted as a function of *β.*

**Figure 2b:**
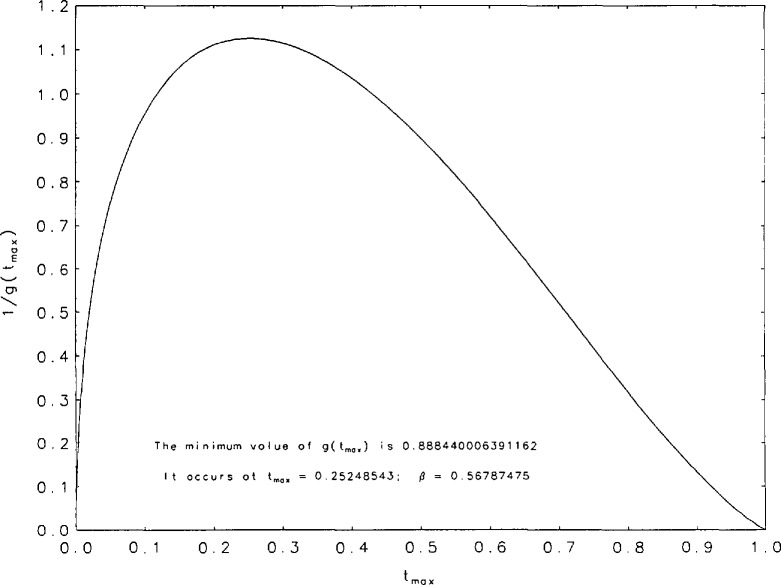
Curve of 1/*g*(*t*_max_) plotted as a function of *t*_max_.

**Table 1:** Relative errors in the numerical inversion of *ĝ*(*s*) for *β* = 1/3, 1/2, and 2/3 for different values of *a* and *T*

	*β*		1/3	1/2	2/3
*a*	*T*	*t*			
2.5	4	0.1	2(−11)	3(−11)	1(−6)
2.5	4	1.0	2(−10)	1(−10)	4(−11)
2.5	4	5.0	6(−10)	5(−10)	4(−10)
2.5	8	0.1	2(−20)	2(−20)	8(−16)
5.0	4	0.01	3(−20)	1(−11)	
5.0	4	0.1	4(−20)	7(−20)	3(−15)
5.0	4	1.0	3(−19)	2(−19)	9(−20)
5.0	4	5.0	1(−18)	1(−18)	8(−19)

**Table 2:** Inverse Laplace transform *g*(*t*) of $\hat{g}\left(s\right)=e^{-s\beta }$

*t*	*β*				
	0.25	0.26	0.27	0.28	0.29
0.00001	0.370151D−04	0.515902D−06	0.248661D−08		
0.00002	0.151806D−02	0.675577D−04	0.144824D−05	0.123255D−07	
0.00003	0.853094D−02	0.649138D−03	0.275324D−04	0.560637D−06	0.451901D−08
0.00004	0.244726D−01	0.258459D−02	0.165894D−03	0.571718D−05	0.902078D−07
0.00005	0.507423D−01	0.672926D−02	0.575158D−03	0.284658D−04	0.711259D−06
0.00006	0.873213D−01	0.137312D−01	0.145291D−02	0.940782D−04	0.330246D−05
0.00007	0.133437D+00	0.239884D−01	0.300022D−02	0.239622D−03	0.109589D−04
0.00008	0.187991D+00	0.376838D−01	0.539788D−02	0.510882D−03	0.289237D−04
0.00009	0.249798D+00	0.548385D−01	0.879472D−02	0.958456D−03	0.647631D−04
0.00010	0.317705D+00	0.753611D−01	0.133042D−01	0.163414D−02	0.128251D−03
0.00011	0.390651D+00	0.990866D−01	0.190057D−01	0.258809D−02	0.231044D−03
0.00012	0.467687D+00	0.125806D+00	0.259484D−01	0.386679D−02	0.386250D−03
0.00013	0.547978D+00	0.155287D+00	0.341556D−01	0.551183D−02	0.607956D−03
0.00014	0.630804D+00	0.187288D+00	0.436295D−01	0.755922D−02	0.910770D−03
0.00015	0.715540D+00	0.221568D+00	0.543553D−01	0.100391D−01	0.130942D−02
0.00016	0.801653D+00	0.257891D+00	0.663051D−01	0.129761D−01	0.181839D−02
0.00017	0.888689D+00	0.296036D+00	0.794409D−01	0.163891D−01	0.245168D−02
0.00018	0.976259D+00	0.335790D+00	0.937176D−01	0.202922D−01	0.322255D−02
0.00019	0.106404D+01	0.376957D+00	0.109085D+00	0.246947D−01	0.414334D−02
0.00020	0.115175D+01	0.419354D+00	0.125488D+00	0.296019D−01	0.522547D−02
0.00021	0.123915D+01	0.462814D+00	0.142873D+00	0.350156D−01	0.647923D−02
0.00022	0.132605D+01	0.507183D+00	0.161181D+00	0.409343D−01	0.791385D−02
0.00023	0.141229D+01	0.552318D+00	0.180357D+00	0.473537D−01	0.953745D−02
0.00024	0.149772D+01	0.598093D+00	0.200343D+00	0.542673D−01	0.113571D−01
0.00025	0.158223D+01	0.644389D+00	0.221084D+00	0.616668D−01	0.133787D−01
0.00030	0.198831D+01	0.880304D+00	0.334310D+00	0.105556D+00	0.266587D−01
0.00035	0.236313D+01	0.111698D+01	0.459197D+00	0.159675D+00	0.453716D−01
0.00040	0.270528D+01	0.134838D+01	0.591057D+00	0.222165D+00	0.694073D−01
0.00045	0.301586D+01	0.157109D+01	0.726376D+00	0.291223D+00	0.984011D−01
0.00050	0.329700D+01	0.178326D+01	0.862582D+00	0.365234D+00	0.131854D+00
0.00060	0.378101D+01	0.217324D+01	0.113082D+01	0.522815D+00	0.209913D+00
0.00070	0.417683D+01	0.251762D+01	0.138668D+01	0.686433D+00	0.299254D+00
0.00080	0.450132D+01	0.281968D+01	0.162607D+01	0.850458D+00	0.396103D+00
0.00090	0.476810D+01	0.308392D+01	0.184753D+01	0.101129D+01	0.497430D+00
0.00100	0.498794D+01	0.331492D+01	0.205105D+01	0.116672D+01	0.600902D+00
0.00110	0.516936D+01	0.351690D+01	0.223731D+01	0.131544D+01	0.704769D+00
0.00120	0.531912D+01	0.369363D+01	0.240740D+01	0.145678D+01	0.807740D+00
0.00130	0.544260D+01	0.384838D+01	0.256249D+01	0.159047D+01	0.908884D+00
0.00140	0.554414D+01	0.398397D+01	0.270380D+01	0.171648D+01	0.100755D+01
0.00150	0.562724D+01	0.410281D+01	0.283253D+01	0.183497D+01	0.110327D+01
0.00160	0.569476D+01	0.420700D+01	0.294978D+01	0.194618D+01	0.119577D+01
0.00170	0.574907D+01	0.429831D+01	0.305657D+01	0.205044D+01	0.128485D+01
0.00180	0.579212D+01	0.437828D+01	0.315385D+01	0.214809D+01	0.137044D+01
0.00190	0.582551D+01	0.444824D+01	0.324247D+01	0.223950D+01	0.145249D+01
0.00200	0.585062D+01	0.450934D+01	0.332320D+01	0.232501D+01	0.153104D+01
0.00210	0.586859D+01	0.456258D+01	0.339676D+01	0.240499D+01	0.160613D+01
0.00220	0.588040D+01	0.460881D+01	0.346376D+01	0.247978D+01	0.167784D+01
0.00230	0.588685D+01	0.464881D+01	0.352477D+01	0.254971D+01	0.174627D+01
0.00240	0.588868D+01	0.468323D+01	0.358032D+01	0.261507D+01	0.181152D+01
0.00250	0.588646D+01	0.471266D+01	0.363086D+01	0.267617D+01	0.187372D+01
0.00300	0.583090D+01	0.480102D+01	0.382187D+01	0.292665D+01	0.214289D+01
0.00350	0.572936D+01	0.482136D+01	0.393647D+01	0.310461D+01	0.235284D+01
0.00400	0.560506D+01	0.480024D+01	0.399971D+01	0.322986D+01	0.251601D+01
0.00450	0.547051D+01	0.475348D+01	0.402773D+01	0.331631D+01	0.264234D+01
0.00500	0.533269D+01	0.469083D+01	0.403120D+01	0.337384D+01	0.273959D+01
0.00600	0.506134D+01	0.454054D+01	0.399133D+01	0.342877D+01	0.286945D+01
0.00700	0.480602D+01	0.437755D+01	0.391576D+01	0.343198D+01	0.293927D+01
0.00800	0.457076D+01	0.421433D+01	0.382270D+01	0.340442D+01	0.296975D+01
0.00900	0.435561D+01	0.405650D+01	0.372195D+01	0.335844D+01	0.297401D+01
0.01000	0.415919D+01	0.390643D+01	0.361892D+01	0.330154D+01	0.296059D+01
0.01100	0.397972D+01	0.376497D+01	0.351666D+01	0.323842D+01	0.293520D+01
0.01200	0.381537D+01	0.363219D+01	0.341686D+01	0.317209D+01	0.290172D+01
0.01300	0.366447D+01	0.350777d+01	0.332044D+01	0.310448D+01	0.286285D+01
0.01400	0.352550D+01	0.339122D+01	0.322787D+01	0.303685D+01	0.282046D+01
0.01500	0.339714D+01	0.328200D+01	0.313931D+01	0.297004D+01	0.277590D+01
0.01600	0.327824D+01	0.317955D+01	0.305479D+01	0.290456D+01	0.273013D+01
0.01700	0.316778D+01	0.308333D+01	0.297421D+01	0.284075D+01	0.268383D+01
0.01800	0.306491D+01	0.299284D+01	0.289744D+01	0.277880D+01	0.263752D+01
0.01900	0.296885D+01	0.290761D+01	0.282430D+01	0.271882D+01	0.259154D+01
0.02000	0.287894D+01	0.282722D+01	0.275460D+01	0.266086D+01	0.254614D+01
0.02100	0.279460D+01	0.275127D+01	0.268816D+01	0.260491D+01	0.250152D+01
0.02200	0.271531D+01	0.267942D+01	0.262478D+01	0.255095D+01	0.245779D+01
0.02300	0.264063D+01	0.261135D+01	0.256428D+01	0.249893D+01	0.241504D+01
0.02400	0.257015D+01	0.254676D+01	0.250649D+01	0.244880D+01	0.237332D+01
0.02500	0.250353D+01	0.248540D+01	0.245125D+01	0.240048D+01	0.233267D+01
0.03000	0.221824D+01	0.221946D+01	0.220816D+01	0.218367D+01	0.214538D+01
0.03500	0.199355D+01	0.200649D+01	0.200950D+01	0.200190D+01	0.198306D+01
0.04000	0.181164D+01	0.183191D+01	0.184418D+01	0.184784D+01	0.184226D+01
0.04500	0.166111D+01	0.168604D+01	0.170445D+01	0.171578D+01	0.171946D+01
0.05000	0.153434D+01	0.156223D+01	0.158475D+01	0.160139D+01	0.161165D+01
0.06000	0.133230D+01	0.136312D+01	0.139020D+01	0.141316D+01	0.143158D+01
0.07000	0.117811D+01	0.120973D+01	0.123868D+01	0.126467D+01	0.128738D+01
0.08000	0.105636D+01	0.108774D+01	0.111719D+01	0.114448D+01	0.116936D+01
0.09000	0.957662D+00	0.988292D+00	0.101751D+01	0.104515D+01	0.107100D+01
0.10000	0.875953D+00	0.905594D+00	0.934205D+00	0.961646D+00	0.987760D+00
0.11000	0.807149D+00	0.835699D+00	0.863499D+00	0.890438D+00	0.916393D+00
0.12000	0.748385D+00	0.775818D+00	0.802711D+00	0.828978D+00	0.854519D+00
0.13000	0.697593D+00	0.723924D+00	0.749875D+00	0.775379D+00	0.800357D+00
0.14000	0.653241D+00	0.678505D+00	0.703514D+00	0.728214D+00	0.752545D+00
0.15000	0.614166D+00	0.638412D+00	0.662499D+00	0.686386D+00	0.710024D+00
0.16000	0.579473D+00	0.602753D+00	0.625950D+00	0.649031D+00	0.671960D+00
0.17000	0.548460D+00	0.570828D+00	0.593171D+00	0.615466D+00	0.637686D+00
0.18000	0.520567D+00	0.542074D+00	0.563604D+00	0.585140D+00	0.606661D+00
0.19000	0.495343D+00	0.516040D+00	0.536798D+00	0.557605D+00	0.578444D+00
0.20000	0.472421D+00	0.492356D+00	0.512382D+00	0.532490D+00	0.552669D+00
0.21000	0.451498D+00	0.470716D+00	0.490048D+00	0.509490D+00	0.529034D+00
0.22000	0.432322D+00	0.450866D+00	0.469541D+00	0.488347D+00	0.507281D+00
0.23000	0.414684D+00	0.432591D+00	0.450645D+00	0.468847D+00	0.487195D+00
0.24000	0.398403D+00	0.415711D+00	0.433176D+00	0.450803D+00	0.468592D+00
0.25000	0.383330D+00	0.400072D+00	0.416980D+00	0.434059D+00	0.451313D+00
0.30000	0.322103D+00	0.336439D+00	0.350960D+00	0.365674D+00	0.380590D+00
0.35000	0.277427D+00	0.289905D+00	0.302564D+00	0.315413D+00	0.328462D+00
0.40000	0.243395D+00	0.254402D+00	0.265577D+00	0.276930D+00	0.288471D+00
0.45000	0.216613D+00	0.226431D+00	0.236401D+00	0.246533D+00	0.256837D+00
0.50000	0.194994D+00	0.203832D+00	0.212807D+00	0.221929D+00	0.231205D+00
0.55000	0.177181D+00	0.185199D+00	0.193341D+00	0.201614D+00	0.210025D+00
0.60000	0.162254D+00	0.169579D+00	0.177013D+00	0.184565D+00	0.192240D+00
0.65000	0.149568D+00	0.156298D+00	0.163126D+00	0.170058D+00	0.177100D+00
0.70000	0.138657D+00	0.144872D+00	0.151174D+00	0.157569D+00	0.164062D+00
0.75000	0.129174D+00	0.134940D+00	0.140783D+00	0.146708D+00	0.152721D+00
0.80000	0.120859D+00	0.126229D+00	0.131668D+00	0.137180D+00	0.142769D+00
0.85000	0.113510D+00	0.118529D+00	0.123610D+00	0.128756D+00	0.133970D+00
0.90000	0.106968D+00	0.111675D+00	0.116437D+00	0.121256D+00	0.126136D+00
0.95000	0.101110D+00	0.105537D+00	0.110012D+00	0.114538D+00	0.119118D+00
1.00000	0.958339D−01	0.100008D+00	0.104226D+00	0.108488D+00	0.112798D+00
1.10000	0.867153D−01	0.904539D−01	0.942257D−01	0.980323D−01	0.101875D+00
1.20000	0.791153D−01	0.824909D−01	0.858919D−01	0.893196D−01	0.927749D−01
1.30000	0.726869D−01	0.757563D−01	0.788447D−01	0.819528D−01	0.850815D−01
1.40000	0.671813D−01	0.699893D−01	0.728108D−01	0.756465D−01	0.784968D−01
1.50000	0.624152D−01	0.649976D−01	0.675892D−01	0.701902D−01	0.728008D−01
1.60000	0.582505D−01	0.606366D−01	0.630282D−01	0.654252D−01	0.678277D−01
1.70000	0.545814D−01	0.567953D−01	0.590116D−01	0.612299D−01	0.634503D−01
1.80000	0.513255D−01	0.533874D−01	0.554483D−01	0.575096D−01	0.595694D−01
1.90000	0.484176D−01	0.503443D−01	0.522683D−01	0.541892D−01	0.561066D−01
2.00000	0.458055D−01	0.476113D−01	0.494124D−01	0.512084D−01	0.529989D−01
2.10000	0.434467D−01	0.451439D−01	0.468347D−01	0.485187D−01	0.501953D−01
2.20000	0.413066D−01	0.429058D−01	0.444971D−01	0.460801D−01	0.476542D−01
2.30000	0.393567D−01	0.408669D−01	0.423681D−01	0.438597D−01	0.453410D−01
2.40000	0.375729D−01	0.390022D−01	0.404214D−01	0.418299D−01	0.432270D−01
2.50000	0.359352D−01	0.372906D−01	0.386350D−01	0.399677D−01	0.412880D−01
2.60000	0.344267D−01	0.357144D−01	0.369902D−01	0.382535D−01	0.395036D−01
2.70000	0.330329D−01	0.342582D−01	0.354710D−01	0.366707D−01	0.378563D−01
2.80000	0.317409D−01	0.329092D−01	0.340640D−01	0.352050D−01	0.363314D−01
2.90000	0.305414D−01	0.316562D−01	0.327573D−01	0.338442D−01	0.349159D−01
3.00000	0.294237D−01	0.304893D−01	0.315408D−01	0.325775D−01	0.335988D−01
3.20000	0.274043D−01	0.283816D−01	0.293441D−01	0.302911D−01	0.312219D−01
3.40000	0.256298D−01	0.265302D−01	0.274154D−01	0.282845D−01	0.291370D−01
3.60000	0.240589D−01	0.248919D−01	0.257093D−01	0.265103D−01	0.272943D−01
3.80000	0.226589D−01	0.234324D−01	0.241901D−01	0.249311D−01	0.256550D−01
4.00000	0.214039D−01	0.221246D−01	0.228292D−01	0.235171D−01	0.241877D−01
4.20000	0.202728D−01	0.209463D−01	0.216036D−01	0.222442D−01	0.228674D−01
4.40000	0.192485D−01	0.198796D−01	0.204945D−01	0.210927D−01	0.216735D−01
4.60000	0.183167D−01	0.189096D−01	0.194863D−01	0.200463D−01	0.205890D−01
4.80000	0.174657D−01	0.180240D−01	0.185661D−01	0.190916D−01	0.196000D−01
5.00000	0.166856D−01	0.172124D−01	0.177231D−01	0.182174D−01	0.186946D−01

*t*	*β*				
	0.30	0.32	0.34	0.36	0.38
0.0001	0.577115D−05	0.121648D−08			
0.0002	0.646619D−03	0.239900D−05			
0.0003	0.513202D−02	0.651487D−04	0.107344D−06		
0.0004	0.173393D−01	0.450932D−03	0.224946D−05		
0.0005	0.392873D−01	0.165274D−02	0.171861D−04	0.212894D−07	
0.0006	0.711617D−01	0.424768D−02	0.750118D−04	0.210542D−06	
0.0007	0.112072D+00	0.875023D−02	0.231320D−03	0.120478D−05	
0.0008	0.160657D+00	0.155375D−01	0.565412D−03	0.478961D−05	0.438912D−08
0.0009	0.215447D+00	0.248343D−01	0.117278D−02	0.147371D−04	0.248393D−07
0.0010	0.275045D+00	0.367290D−01	0.215567D−02	0.375741D−04	0.104716D−06
0.0011	0.338203D+00	0.512025D−01	0.361447D−02	0.830781D−04	0.353443D−06
0.0012	0.403853D+00	0.681562D−01	0.564119D−02	0.164407D−03	0.100396D−05
0.0013	0.471095D+00	0.874382D−01	0.831519D−02	0.297882D−03	0.248718D−05
0.0014	0.539192D+00	0.108863D+00	0.117010D−01	0.502496D−03	0.551702D−05
0.0015	0.607539D+00	0.132228D+00	0.158477D−01	0.799254D−03	0.111767D−04
0.0016	0.675651D+00	0.157324D+00	0.207892D−01	0.121045D−02	0.209968D−04
0.0017	0.743142D+00	0.183942D+00	0.265457D−01	0.175892D−02	0.370175D−04
0.0018	0.809706D+00	0.211881D+00	0.331246D−01	0.246740D−02	0.618297D−04
0.0019	0.875106D+00	0.240950D+00	0.405229D−01	0.335793D−02	0.985926D−04
0.0020	0.939159D+00	0.270970D+00	0.487283D−01	0.445136D−02	0.151029D−03
0.0021	0.100173D+01	0.301776D+00	0.577210D−01	0.576702D−02	0.223396D−03
0.0022	0.106272D+01	0.333216D+00	0.674750D−01	0.732241D−02	0.320443D−03
0.0023	0.112206D+01	0.365153D+00	0.779600D−01	0.913304D−02	0.447353D−03
0.0024	0.117970D+01	0.397460D+00	0.891418D−01	0.112123D−01	0.609668D−03
0.0025	0.123562D+01	0.430026D+00	0.100984D+00	0.135716D−01	0.813220D−03
0.0030	0.148946D+01	0.593555D+00	0.168732D+00	0.298190D−01	0.266390D−02
0.0035	0.170271D+01	0.752371D+00	0.247165D+00	0.536557D−01	0.645439D−02
0.0040	0.188023D+01	0.901685D+00	0.331963D+00	0.846472D−01	0.128304D−01
0.0045	0.202727D+01	0.103934D+Ò1	0.419697D+00	0.121852D+00	0.222298D−01
0.0050	0.214868D+01	0.116469D+01	0.507819D+00	0.164122D+00	0.348631D−01
0.0060	0.233062D+01	0.137960D+01	0.678587D+00	0.259259D+00	0.697247D−01
0.0070	0.245192D+01	0.155178D+01	0.835891D+00	0.361944D+00	0.115960D+00
0.0080	0.253036D+01	0.168824D+01	0.976603D+00	0.466212D+00	0.171035D+00
0.0090	0.257814D+01	0.179553D+01	0.110029D+01	0.568107D+00	0.232237D+00
0.0100	0.260376D+01	0.187922D+01	0.120782D+01	0.665204D+00	0.297120D+00
0.0110	0.261321D+01	0.194384D+01	0.130058D+01	0.756146D+00	0.363668D+00
0.0120	0.261078D+01	0.199304D+01	0.138014D+01	0.840293D+00	0.430308D+00
0.0130	0.259958D+01	0.202974D+01	0.144806D+01	0.917463D+00	0.495866D+00
0.0140	0.258189D+01	0.205627D+01	0.150576D+01	0.987763D+00	0.559498D+00
0.0150	0.255944D+01	0.207450D+01	0.155455D+01	0.105147D+01	0.620622D+00
0.0160	0.253349D+01	0.208594D+01	0.159556D+01	0.110897D+01	0.678861D+00
0.0170	0.250502D+01	0.209182D+01	0.162981D+01	0.116067D+01	0.733992D+00
0.0180	0.247477D+01	0.209311D+01	0.165817D+01	0.120701D+01	0.785906D+00
0.0190	0.244331D+01	0.209064D+01	0.168140D+01	0.124843D+01	0.834581D+00
0.0200	0.241107D+01	0.208507D+01	0.170017D+01	0.128534D+01	0.880053D+00
0.0210	0.237838D+01	0.207695D+01	0.171503D+01	0.131813D+01	0.922401D+00
0.0220	0.234552D+01	0.206673D+01	0.172649D+01	0.134716D+01	0.961733D+00
0.0230	0.231268D+01	0.205478D+01	0.173499D+01	0.137279D+01	0.998177D+00
0.0240	0.228001D+01	0.204143D+01	0.174088D+01	0.139531D+01	0.103187D+01
0.0250	0.224765D+01	0.202693D+01	0.174450D+01	0.141501D+01	0.106296D+01
0.0260	0.221567D+01	0.201150D+01	0.174613D+01	0.143214D+01	0.109158D+01
0.0270	0.218416D+01	0.199533D+01	0.174602D+01	0.144695D+01	0.111789D+01
0.0280	0.215315D+01	0.197858D+01	0.174439D+01	0.145965D+01	0.114203D+01
0.0290	0.212269D+01	0.196137D+01	0.174142D+01	0.147043D+01	0.116412D+01
0.0300	0.209281D+01	0.194381D+01	0.173728D+01	0.147947D+01	0.118430D+01
0.0310	0.206352D+01	0.192601D+01	0.173212D+01	0.148692D+01	0.120269D+01
0.0320	0.203482D+01	0.190803D+01	0.172607D+01	0.149294D+01	0.121942D+01
0.0330	0.200674D+01	0.188995D+01	0.171924D+01	0.149765D+01	0.123458D+01
0.0340	0.197927D+01	0.187183D+01	0.171172D+01	0.150117D+01	0.124830D+01
0.0350	0.195239D+01	0.185370D+01	0.170362D+01	0.150361D+01	0.126065D+01
0.0360	0.192612D+01	0.183561D+01	0.169501D+01	0.150508D+01	0.127175D+01
0.0370	0.190044D+01	0.181760D+01	0.168595D+01	0.150565D+01	0.128166D+01
0.0380	0.187534D+01	0.179970D+01	0.167651D+01	0.150541D+01	0.129048D+01
0.0390	0.185081D+01	0.178192D+01	0.166675D+01	0.150444D+01	0.129828D+01
0.0400	0.182684D+01	0.176430D+01	0.165670D+01	0.150279D+01	0.130512D+01
0.0410	0.180341D+01	0.174684D+01	0.164643D+01	0.150054D+01	0.131108D+01
0.0420	0.178052D+01	0.172956D+01	0.163595D+01	0.149774D+01	0.131621D+01
0.0430	0.175815D+01	0.171247D+01	0.162532D+01	0.149444D+01	0.132058D+01
0.0440	0.173628D+01	0.169558D+01	0.161455D+01	0.149068D+01	0.132422D+01
0.0450	0.171491D+01	0.167890D+01	0.160368D+01	0.148651D+01	0.132720D+01
0.0460	0.169402D+01	0.166243D+01	0.159273D+01	0.148197D+01	0.132956D+01
0.0470	0.167359D+01	0.164618D+01	0.158172D+01	0.147710D+01	0.133134D+01
0.0480	0.165362D+01	0.163015D+01	0.157067D+01	0.147191D+01	0.133258D+01
0.0490	0.163410D+01	0.161435D+01	0.155960D+01	0.146646D+01	0.133332D+01
0.0500	0.161500D+01	0.159877D+01	0.154852D+01	0.146075D+01	0.133359D+01
0.0600	0.144504D+01	0.145517D+01	0.143969D+01	0.139471D+01	0.131684D+01
0.0700	0.130645D+01	0.133216D+01	0.133847D+01	0.132177D+01	0.127837D+01
0.0800	0.119155D+01	0.122661D+01	0.124690D+01	0.124924D+01	0.123015D+01
0.0900	0.109484D+01	0.113550D+01	0.116485D+01	0.118018D+01	0.117840D+01
0.1000	0.101237D+01	0.105627D+01	0.109149D+01	0.111576D+01	0.112640D+01
0.1100	0.941223D+00	0.986854D+00	0.102582D+01	0.105624D+01	0.107582D+01
0.1200	0.879225D+00	0.925600D+00	0.966874D+00	0.100149D+01	0.102752D+01
0.1300	0.824722D+00	0.871187D+00	0.913776D+00	0.951209D+00	0.981859D+00
0.1400	0.776435D+00	0.822554D+00	0.865765D+00	0.905016D+00	0.938950D+00
0.1500	0.733358D+00	0.778843D+00	0.822190D+00	0.862536D+00	0.898755D+00
0.1600	0.694693D+00	0.739355D+00	0.782493D+00	0.823407D+00	0.861163D+00
0.1700	0.659795D+00	0.703513D+00	0.746203D+00	0.787296D+00	0.826024D+00
0.1800	0.628140D+00	0.670841D+00	0.712914D+00	0.753901D+00	0.793174D+00
0.1900	0.599296D+00	0.640941D+00	0.682282D+00	0.722953D+00	0.762444D+00
0.2000	0.572906D+00	0.613478D+00	0.654009D+00	0.694212D+00	0.733674D+00
0.2100	0.548670D+00	0.588169D+00	0.627842D+00	0.667464D+00	0.706710D+00
0.2200	0.526336D+00	0.564774D+00	0.603558D+00	0.642522D+00	0.681408D+00
0.2300	0.505687D+00	0.543084D+00	0.580968D+00	0.619217D+00	0.657637D+00
0.2400	0.486542D+00	0.522922D+00	0.559902D+00	0.597401D+00	0.635274D+00
0.2500	0.468742D+00	0.504133D+00	0.540216D+00	0.576943D+00	0.614211D+00
0.3000	0.395716D+00	0.426630D+00	0.458474D+00	0.491299D+00	0.525137D+00
0.3500	0.341722D+00	0.368917D+00	0.397083D+00	0.426309D+00	0.456686D+00
0.4000	0.300210D+00	0.324328D+00	0.349379D+00	0.375462D+00	0.402687D+00
0.4500	0.267323D+00	0.288884D+00	0.311305D+00	0.334687D+00	0.359140D+00
0.5000	0.240646D+00	0.260059D+00	0.280252D+00	0.301319D+00	0.323364D+00
0.5500	0.218584D+00	0.236178D+00	0.254471D+00	0.273549D+00	0.293507D+00
0.6000	0.200046D+00	0.216084D+00	0.232746D+00	0.250107D+00	0.268253D+00
0.6500	0.184259D+00	0.198954D+00	0.214204D+00	0.230075D+00	0.246642D+00
0.7000	0.170658D+00	0.184187D+00	0.198206D+00	0.212776D+00	0.227960D+00
0.7500	0.158825D+00	0.171331D+00	0.184272D+00	0.197699D+00	0.211668D+00
0.8000	0.148440D+00	0.160045D+00	0.172034D+00	0.184451D+00	0.197345D+00
0.8500	0.139256D+00	0.150062D+00	0.161206D+00	0.172727D+00	0.184667D+00
0.9000	0.131079D+00	0.141172D+00	0.151563D+00	0.162285D+00	0.173373D+00
0.9500	0.123754D+00	0.133208D+00	0.142923D+00	0.152929D+00	0.163256D+00
1.0000	0.117157D+00	0.126035D+00	0.135143D+00	0.144504D+00	0.154145D+00
1.1000	0.105757D+00	0.113641D+00	0.121701D+00	0.129953D+00	0.138414D+00
1.2000	0.962591D−01	0.103319D+00	0.110511D+00	0.117844D+00	0.125330D+00
1.3000	0.882314D−01	0.945984D−01	0.101060D+00	0.107624D+00	0.114296D+00
1.4000	0.813620D−01	0.871394D−01	0.929821D−01	0.988936D−01	0.104877D+00
1.5000	0.754212D−01	0.806920D−01	0.860039D−01	0.913578D−01	0.967540D−01
1.6000	0.702356D−01	0.750676D−01	0.799205D−01	0.847932D−01	0.896840D−01
1.7000	0.656724D−01	0.701212D−01	0.745741D−01	0.790284D−01	0.834810D−01
1.8000	0.616280D−01	0.657397D−01	0.698416D−01	0.739298D−01	0.779998D−01
1.9000	0.580202D−01	0.618337D−01	.0.656257D−01	0.693913D−01	0.731251D−01
2.0000	0.547832D−01	0.583314D−01	0.618482D−01	0.653281D−01	0.687650D−01
2.1000	0.518640D−01	0.551748D−01	0.584460D−01	0.616714D−01	0.648445D−01
2.2000	0.492187D−01	0.523163D−01	0.553672D−01	0.583650D−01	0.613027D−01
2.3000	0.468114D−01	0.497165D−01	0.525690D−01	0.553622D−01	0.580890D−01
2.4000	0.446119D−01	0.473426D−01	0.500157D−01	0.526245D−01	0.551615D−01
2.5000	0.425952D−01	0.451673D−01	0.476776D−01	0.501192D−01	0.524848D−01
2.6000	0.407397D−01	0.431671D−01	0.455291D−01	0.478190D−01	0.500291D−01
2.7000	0.390273D−01	0.413222D−01	0.435489D−01	0.457003D−01	0.477692D−01
2.8000	0.374425D−01	0.396158D−01	0.417184D−01	0.437433D−01	0.456833D−01
2.9000	0.359719D−01	0.380332D−01	0.400218D−01	0.419307D−01	0.437529D−01
3.0000	0.346037D−01	0.365617D−01	0.384453D−01	0.402476D−01	0.419617D−01
3.1000	0.333279D−01	0.351904D−01	0.369770D−01	0.386811D−01	0.402959D−01
3.2000	0.321358D−01	0.339096D−01	0.356065D−01	0.372199D−01	0.387431D−01
3.3000	0.310194D−01	0.327109D−01	0.343246D−01	0.358540D−01	0.372927D−01
3.4000	0.299720D−01	0.315869D−01	0.331232D−01	0.345748D−01	0.359353D−01
3.5000	0.289875D−01	0.305309D−01	0.319952D−01	0.333745D−01	0.346625D−01
3.6000	0.280606D−01	0.295372D−01	0.309344D−01	0.322463D−01	0.334670D−01
3.7000	0.271865D−01	0.286006D−01	0.299350D−01	0.311841D−01	0.323421D−01
3.8000	0.263609D−01	0.277164D−01	0.289921D−01	0.301825D−01	0.312821D−01
3.9000	0.255800D−01	0.268804D−01	0.281011D−01	0.292366D−01	0.302817D−01
4.0000	0.248403D−01	0.260890D−01	0.272580D−01	0.283421D−01	0.293363D−01
4.2000	0.234726D−01	0.246266D−01	0.257013D−01	0.266919D−01	0.275936D−01
4.4000	0.222363D−01	0.233060D−01	0.242970D−01	0.252048D−01	0.260250D−01
4.6000	0.211139D−01	0.221080D−01	0.230243D−01	0.238585D−01	0.246066D−01
4.8000	0.200907D−01	0.210169D−01	0.218662D−01	0.226346D−01	0.233185D−01
5.0000	0.191544D−01	0.200192D−01	0.208083D−01	0.215177D−01	0.221443D−01

*t*	*β*				
	0.40	0.42	0.44	0.46	0.48
0.002	0.116562D−05				
0.003	0.875664D−04	0.642966D−06			
0.004	0.917142D−03	0.216038D−04	0.949439D−07		
0.005	0.406492D−02	0.197437D−03	0.259248D−05	0.458884D−08	
0.006	0.114034D−01	0.908478D−03	0.249859D−04	0.137126D−06	
0.007	0.243242D−01	0.277903D−02	0.130277D−03	0.160298D−05	0.250858D−08
0.008	0.434357D−01	0.653168D−02	0.458125D−03	0.103165D−04	0.409163D−07
0.009	0.686419D−01	0.128176D−01	0.123210D−02	0.443480D−04	0.359676D−06
0.010	0.993632D−01	0.221063D−01	0.273747D−02	0.143247D−03	0.204689D−05
0.011	0.134755D+00	0.346471D−01	0.528212D−02	0.375074D−03	0.848024D−05
0.012	0.173873D+00	0.504823D−01	0.915677D−02	0.837893D−03	0.276694D−04
0.013	0.215782D+00	0.694867D−01	0.146046D−01	0.165512D−02	0.750945D−04
0.014	0.259618D+00	0.914136D−01	0.218037D−01	0.296638D−02	0.176300D−03
0.015	0.304618D+00	0.115939D+00	0.308610D−01	0.491645D−02	0.368510D−03
0.016	0.350132D+00	0.142698D+00	0.418152D−01	0.764441D−02	0.700833D−03
0.017	0.395618D+00	0.171312D+00	0.546444D−01	0.112748D−01	0.123301D−02
0.018	0.440637D+00	0.201408D+00	0.692772D−01	0.159112D−01	0.203294D−02
0.019	0.484838D+00	0.232634D+00	0.856035D−01	0.216325D−01	0.317351D−02
0.020	0.527948D+00	0.264664D+00	0.103486D+00	0.284915D−01	0.472905D−02
0.021	0.569763D+00	0.297204D+00	0.122768D+00	0.365153D−01	0.677197D−02
0.022	0.610129D+00	0.329992D+00	0.143285D+00	0.457071D−01	0.936977D−02
0.023	0.648942D+00	0.362802D+00	0.164867D+00	0.560491D−01	0.125826D−01
0.024	0.686132D+00	0.395437D+00	0.187346D+00	0.675050D−01	0.164616D−01
0.025	0.721660D+00	0.427728D+00	0.210559D+00	0.800237D−01	0.210476D−01
0.026	0.755512D+00	0.459537D+00	0.234352D+00	0.935423D−01	0.263709D−01
0.027	0.787691D+00	0.490746D+00	0.258579D+00	0.107989D+00	0.324507D−01
0.028	0.818217D+00	0.521260D+00	0.283106D+00	0.123286D+00	0.392962D−01
0.029	0.847120D+00	0.551004D+00	0.307810D+00	0.139352D+00	0.469066D−01
0.030	0.874438D+00	0.579917D+00	0.332580D+00	0.156103D+00	0.552723D−01
0.031	0.900219D+00	0.607954D+00	0.357315D+00	0.173454D+00	0.643757D−01
0.032	0.924511D+00	0.635082D+00	0.381927D+00	0.191325D+00	0.741927D−01
0.033	0.947368D+00	0.661279D+00	0.406335D+00	0.209633D+00	0.846930D−01
0.034	0.968846D+00	0.686532D+00	0.430472D+00	0.228302D+00	0.958419D−01
0.035	0.989000D+00	0.710833D+00	0.454277D+00	0.247258D+00	0.107601D+00
0.036	0.100789D+01	0.734185D+00	0.477698D+00	0.266430D+00	0.119928D+00
0.037	0.102556D+01	0.756592D+00	0.500691D+00	0.285755D+00	0.132781D+00
0.038	0.104208D+01	0.778065D+00	0.523220D+00	0.305170D+00	0.146115D+00
0.039	0.105750D+01	0.798618D+00	0.545253D+00	0.324619D+00	0.159883D+00
0.040	0.107186D+01	0.818267D+00	0.566765D+00	0.344050D+00	0.174042D+00
0.041	0.108523D+01	0.837032D+00	0.587736D+00	0.363416D+00	0.188546D+00
0.042	0.109765D+01	0.854934D+00	0.608151D+00	0.382673D+00	0.203351D+00
0.043	0.110916D+01	0.871994D+00	0.627997D+00	0.401783D+00	0.218414D+00
0.044	0.111982D+01	0.888237D+00	0.647268D+00	0.420710D+00	0.233694D+00
0.045	0.112967D+01	0.903687D+00	0.665957D+00	0.439423D+00	0.249150D+00
0.046	0.113875D+01	0.918367D+00	0.684064D+00	0.457894D+00	0.264745D+00
0.047	0.114709D+01	0.932303D+00	0.701587D+00	0.476097D+00	0.280442D+00
0.048	0.115474D+01	0.945520D+00	0.718531D+00	0.494012D+00	0.296207D+00
0.049	0.116173D+01	0.958043D+00	0.734898D+00	0.511619D+00	0.312007D+00
0.050	0.116810D+01	0.969895D+00	0.750694D+00	0.528903D+00	0.327812D+00
0.060	0.120401D+01	0.105666D+01	0.879260D+00	0.681859D+00	0.480848D+00
0.070	0.120500D+01	0.109958D+01	0.962365D+00	0.797412D+00	0.614166D+00
0.080	0.118608D+01	0.111388D+01	0.101155D+01	0.879415D+00	0.721629D+00
0.090	0.115610D+01	0.110979D+01	0.103644D+01	0.934203D+00	0.803710D+00
0.100	0.112030D+01	0.109406D+01	0.104425D+01	0.967935D+00	0.863552D+00
0.110	0.108183D+01	0.107110D+01	0.104023D+01	0.985781D+00	0.904981D+00
0.120	0.104258D+01	0.104386D+01	0.102810D+01	0.991828D+00	0.931641D+00
0.130	0.100370D+01	0.101424D+01	0.101054D+01	0.989212D+00	0.946687D+00
0.140	0.965844D+00	0.983542D+00	0.989407D+00	0.980311D+00	0.952723D+00
0.150	0.929393D+00	0.952596D+00	0.966045D+00	0.966908D+00	0.951843D+00
0.160	0.894540D+00	0.921958D+00	0.941400D+00	0.950341D+00	0.945708D+00
0.170	0.861368D+00	0.891987D+00	0.916142D+00	0.931613D+00	0.935623D+00
0.180	0.829885D+00	0.862904D+00	0.890744D+00	0.911470D+00	0.922610D+00
0.190	0.800057D+00	0.834843D+00	0.865538D+00	0.890471D+00	0.907465D+00
0.200	0.771825D+00	0.807874D+00	0.840754D+00	0.869031D+00	0.890808D+00
0.210	0.745114D+00	0.782026D+00	0.816548D+00	0.847460D+00	0.873120D+00
0.220	0.719845D+00	0.757298D+00	0.793022D+00	0.825984D+00	0.854775D+00
0.230	0.695932D+00	0.733672D+00	0.770239D+00	0.804769D+00	0.836061D+00
0.240	0.673294D+00	0.711115D+00	0.748236D+00	0.783936D+00	0.817201D+00
0.250	0.651849D+00	0.689590D+00	0.727027D+00	0.763569D+00	0.798366D+00
0.260	0.631522D+00	0.669052D+00	0.706614D+00	0.743726D+00	0.779685D+00
0.270	0.612240D+00	0.649456D+00	0.686986D+00	0.724445D+00	0.761256D+00
0.280	0.593933D+00	0.630755D+00	0.668127D+00	0.705748D+00	0.743153D+00
0.290	0.576540D+00	0.612903D+00	0.650014D+00	0.687645D+00	0.725428D+00
0.300	0.559999D+00	0.595856D+00	0.632624D+00	0.670140D+00	0.708119D+00
0.310	0.544257D+00	0.579569D+00	0.615929D+00	0.653227D+00	0.691253D+00
0.320	0.529261D+00	0.564002D+00	0.599901D+00	0.636897D+00	0.674844D+00
0.330	0.514965D+00	0.549114D+00	0.584513D+00	0.621139D+00	0.658903D+00
0.340	0.501325D+00	0.534868D+00	0.569735D+00	0.605937D+00	0.643431D+00
0.350	0.488299D+00	0.521229D+00	0.555540D+00	0.591274D+00	0.628428D+00
0.360	0.475851D+00	0.508163D+00	0.541902D+00	0.577132D+00	0.613889D+00
0.370	0.463945D+00	0.495639D+00	0.528793D+00	0.563494D+00	0.599805D+00
0.380	0.452549D+00	0.483627D+00	0.516190D+00	0.550341D+00	0.586169D+00
0.390	0.441633D+00	0.472100D+00	0.504067D+00	0.537653D+00	0.572969D+00
0.400	0.431169D+00	0.461031D+00	0.492402D+00	0.525413D+00	0.560193D+00
0.410	0.421131D+00	0.450396D+00	0.481173D+00	0.513603D+00	0.547829D+00
0.420	0.411496D+00	0.440173D+00	0.470359D+00	0.502205D+00	0.535864D+00
0.430	0.402240D+00	0.430339D+00	0.459941D+00	0.491201D+00	0.524285D+00
0.440	0.393343D+00	0.420875D+00	0.449899D+00	0.480575D+00	0.513078D+00
0.450	0.384786D+00	0.411762D+00	0.440216D+00	0.470312D+00	0.502232D+00
0.460	0.376551D+00	0.402982D+00	0.430874D+00	0.460396D+00	0.491732D+00
0.470	0.368621D+00	0.394518D+00	0.421859D+00	0.450812D+00	0.481566D+00
0.480	0.360979D+00	0.386355D+00	0.413154D+00	0.441546D+00	0.471721D+00
0.490	0.353612D+00	0.378479D+00	0.404747D+00	0.432585D+00	0.462187D+00
0.500	0.346505D+00	0.370874D+00	0.396622D+00	0.423916D+00	0.452950D+00
0.550	0.314454D+00	0.336511D+00	0.359819D+00	0.384538D+00	0.410854D+00
0.600	0.287281D+00	0.307300D+00	0.328438D+00	0.350840D+00	0.374675D+00
0.650	0.263990D+00	0.282217D+00	0.301435D+00	0.321772D+00	0.343378D+00
0.700	0.243835D+00	0.260483D+00	0.278004D+00	0.296508D+00	0.316126D+00
0.750	0.226243D+00	0.241498D+00	0.257517D+00	0.274396D+00	0.292247D+00
0.800	0.210772D+00	0.224794D+00	0.239482D+00	0.254918D+00	0.271198D+00
0.850	0.197073D+00	0.209999D+00	0.223504D+00	0.237658D+00	0.252541D+00
0.900	0.184869D+00	0.196817D+00	0.209268D+00	0.222279D+00	0.235919D+00
0.950	0.173937D+00	0.185010D+00	0.196517D+00	0.208508D+00	0.221036D+00
1.000	0.164093D+00	0.174381D+00	0.185042D+00	0.196118D+00	0.207652D+00
1.100	0.147104D+00	0.156044D+00	0.165256D+00	0.174768D+00	0.184607D+00
1.200	0.132983D+00	0.140815D+00	0.148840D+00	0.157075D+00	0.165535D+00
1.300	0.121084D+00	0.127994D+00	0.135036D+00	0.142215D+00	0.149542D+00
1.400	0.110936D+00	0.117073D+00	0.123290D+00	0.129591D+00	0.135976D+00
1.500	0.102193D+00	0.107673D+00	0.113195D+00	0.118755D+00	0.124352D+00
1.600	0.945906D−01	0.995101D−01	0.104439D+00	0.109371D+00	0.114302D+00
1.700	0.879277D−01	0.923638D−01	0.967832D−01	0.101179D+00	0.105542D+00
1.800	0.820461D−01	0.860627D−01	0.900420D−01	0.939757D−01	0.978536D−01
1.900	0.768208D−01	0.804712D−01	0.840678D−01	0.876011D−01	0.910602D−01
2.000	0.721517D−01	0.754805D−01	0.787423D−01	0.819268D−01	0.850226D−01
2.100	0.679577D−01	0.710027D−01	0.739700D−01	0.768490D−01	0.796279D−01
2.200	0.641725D−01	0.669658D−01	0.696728D−01	0.722829D−01	0.747841D−01
2.300	0.607413D−01	0.633104D−01	0.657863D−01	0.681586D−01	0.704153D−01
2.400	0.576186D−01	0.599870D−01	0.622571D−01	0.644182D−01	0.664587D−01
2.500	0.547661D−01	0.569544D−01	0.590402D−01	0.610130D−01	0.628616D−01
2.600	0.521515D−01	0.541776D−01	0.560979D−01	0.579023D−01	0.595799D−01
2.700	0.497475D−01	0.516269D−01	0.533980D−01	0.550513D−01	0.565761D−01
2.800	0.475306D−01	0.492769D−01	0.509133D−01	0.524304D−01	0.538181D−01
2.900	0.454806D−01	0.471059D−01	0.486201D−01	0.500143D−01	0.512787D−01
3.000	0.435802D−01	0.450951D−01	0.464983D−01	0.477811D−01	0.489343D−01
3.100	0.418141D−01	0.432281D−01	0.445301D−01	0.457118D−01	0.467645D−01
3.200	0.401692D−01	0.414908D−01	0.427004D−01	0.437900D−01	0.447516D−01
3.300	0.386340D−01	0.398707D−01	0.409956D−01	0.420013D−01	0.428800D−01
3.400	0.371982D−01	0.383568D−01	0.394041D−01	0.403330D−01	0.411363D−01
3.500	0.358530D−01	0.369395D−01	0.379154D−01	0.387740D−01	0.395084D−01
3.600	0.345904D−01	0.356103D−01	0.365204D−01	0.373145D−01	0.379860D−01
3.700	0.334032D−01	0.343615D−01	0.352110D−01	0.359457D−01	0.365595D−01
3.800	0.322853D−01	0.331864D−01	0.339798D−01	0.346598D−01	0.352208D−01
3.900	0.312310D−01	0.320790D−01	0.328205D−01	0.334500D−01	0.339624D−01
4.000	0.302352D−01	0.310339D−01	0.317272D−01	0.323101D−01	0.327778D−01
4.100	0.292935D−01	0.300461D−01	0.306947D−01	0.312345D−01	0.316609D−01
4.200	0.284016D−01	0.291114D−01	0.297184D−01	0.302182D−01	0.306065D−01
4.300	0.275560D−01	0.282257D−01	0.287939D−01	0.292566D−01	0.296098D−01
4.400	0.267533D−01	0.273855D−01	0.279176D−01	0.283458D−01	0.286664D−01
4.500	0.259904D−01	0.265875D−01	0.270859D−01	0.274820D−01	0.277725D−01
4.600	0.252646D−01	0.258288D−01	0.262956D−01	0.266618D−01	0.269243D−01
4.700	0.245733D−01	0.251066D−01	0.255440D−01	0.258823D−01	0.261188D−01
4.800	0.239143D−01	0.244186D−01	0.248283D−01	0.251406D−01	0.253530D−01
4.900	0.232854D−01	0.237624D−01	0.241462D−01	0.244342D−01	0.246241D−01
5.000	0.226847D−01	0.231360D−01	0.234955D−01	0.237608D−01	0.239298D−01

*t*	*β*				
	0.50	0.52	0.54	0.56	0.58
0.01	0.391772D−08				
0.02	0.371680D−03	0.939964D−05	0.404234D−07		
0.03	0.130495D−01	0.169605D−02	0.893449D−04	0.115097D−05	0.152735D−08
0.04	0.680714D−01	0.183678D−01	0.286174D−02	0.193321D−03	0.351487D−05
0.05	0.170007D+00	0.684450D−01	0.190661D−01	0.306418D−02	0.211287D−03
0.06	0.297583D+00	0.153336D+00	0.607468D−01	0.163477D−01	0.245053D−02
0.07	0.428249D+00	0.260102D+00	0.129875D+00	0.487872D−01	0.119843D−01
0.08	0.547759D+00	0.373510D+00	0.219075D+00	0.103472D+00	0.355059D−01
0.09	0.649618D+00	0.482197D+00	0.317871D+00	0.176950D+00	0.769517D−01
0.10	0.732249D+00	0.579576D+00	0.417048D+00	0.262290D+00	0.135746D+00
0.11	0.796660D+00	0.662765D+00	0.510177D+00	0.352173D+00	0.207879D+00
0.12	0.844973D+00	0.731240D+00	0.593536D+00	0.440582D+00	0.287906D+00
0.13	0.879627D+00	0.785795D+00	0.665456D+00	0.523313D+00	0.370513D+00
0.14	0.902978D+00	0.827855D+00	0.725645D+00	0.597843D+00	0.451359D+00
0.15	0.917137D+00	0.859059D+00	0.774628D+00	0.662958D+00	0.527319D+00
0.16	0.923911D+00	0.881041D+00	0.813371D+00	0.718366D+00	0.596414D+00
0.17	0.924812D+00	0.895307D+00	0.843025D+00	0.764370D+00	0.657596D+00
0.18	0.921085D+00	0.903197D+00	0.864775D+00	0.801619D+00	0.710505D+00
0.19	0.913744D+00	0.905866D+00	0.879753D+00	0.830941D+00	0.755259D+00
0.20	0.903612D+00	0.904299D+00	0.889000D+00	0.853224D+00	0.792275D+00
0.21	0.891351D+00	0.899321D+00	0.893441D+00	0.869353D+00	0.822150D+00
0.22	0.877495D+00	0.891623D+00	0.893884D+00	0.880163D+00	0.845564D+00
0.23	0.862470D+00	0.881775D+00	0.891029D+00	0.886419D+00	0.863221D+00
0.24	0.846619D+00	0.870251D+00	0.885473D+00	0.888811D+00	0.875812D+00
0.25	0.830215D+00	0.857438D+00	0.877724D+00	0.887948D+00	0.883987D+00
0.26	0.813475D+00	0.843656D+00	0.868212D+00	0.884361D+00	0.888347D+00
0.27	0.796572D+00	0.829167D+00	0.857297D+00	0.878513D+00	0.889435D+00
0.28	0.779642D+00	0.814183D+00	0.845282D+00	0.870802D+00	0.887738D+00
0.29	0.762792D+00	0.798880D+00	0.832421D+00	0.861571D+00	0.883684D+00
0.30	0.746107D+00	0.783398D+00	0.818926D+00	0.851113D+00	0.877653D+00
0.31	0.729651D+00	0.767851D+00	0.804972D+00	0.839675D+00	0.869973D+00
0.32	0.713473D+00	0.752334D+00	0.790705D+00	0.827470D+00	0.860931D+00
0.33	0.697611D+00	0.736918D+00	0.776247D+00	0.814677D+00	0.850774D+00
0.34	0.682091D+00	0.721663D+00	0.761697D+00	0.801446D+00	0.839717D+00
0.35	0.666934D+00	0.706617D+00	0.747138D+00	0.787906D+00	0.827944D+00
0.36	0.652151D+00	0.691813D+00	0.732636D+00	0.774163D+00	0.815611D+00
0.37	0.637750D+00	0.677282D+00	0.718246D+00	0.760308D+00	0.802854D+00
0.38	0.623734D+00	0.663043D+00	0.704012D+00	0.746414D+00	0.789787D+00
0.39	0.610105D+00	0.649111D+00	0.689970D+00	0.732546D+00	0.776508D+00
0.40	0.596858D+00	0.635497D+00	0.676147D+00	0.718753D+00	0.763100D+00
0.41	0.583990D+00	0.622208D+00	0.662566D+00	0.705078D+00	0.749633D+00
0.42	0.571495D+00	0.609246D+00	0.649243D+00	0.691557D+00	0.736166D+00
0.43	0.559365D+00	0.596614D+00	0.636190D+00	0.678217D+00	0.722748D+00
0.44	0.547593D+00	0.584310D+00	0.623416D+00	0.665081D+00	0.709421D+00
0.45	0.536169D+00	0.572330D+00	0.610928D+00	0.652166D+00	0.696218D+00
0.46	0.525084D+00	0.560672D+00	0.598728D+00	0.639487D+00	0.683169D+00
0.47	0.514328D+00	0.549330D+00	0.586818D+00	0.627053D+00	0.670296D+00
0.48	0.503893D+00	0.538298D+00	0.575198D+00	0.614873D+00	0.657618D+00
0.49	0.493767D+00	0.527569D+00	0.563865D+00	0.602951D+00	0.645149D+00
0.50	0.483941D+00	0.517138D+00	0.552817D+00	0.591291D+00	0.632902D+00
0.51	0.474406D+00	0.506995D+00	0.542050D+00	0.579894D+00	0.620885D+00
0.52	0.465152D+00	0.497134D+00	0.531560D+00	0.568760D+00	0.609105D+00
0.53	0.456169D+00	0.487547D+00	0.521342D+00	0.557887D+00	0.597566D+00
0.54	0.447448D+00	0.478227D+00	0.511390D+00	0.547275D+00	0.586270D+00
0.55	0.438980D+00	0.469165D+00	0.501699D+00	0.536919D+00	0.575220D+00
0.56	0.430756D+00	0.460354D+00	0.492261D+00	0.526816D+00	0.564416D+00
0.57	0.422769D+00	0.451786D+00	0.483072D+00	0.516963D+00	0.553855D+00
0.58	0.415009D+00	0.443454D+00	0.474125D+00	0.507354D+00	0.543538D+00
0.59	0.407468D+00	0.435350D+00	0.465413D+00	0.497985D+00	0.533461D+00
0.60	0.400140D+00	0.427467D+00	0.456929D+00	0.488851D+00	0.523621D+00
0.61	0.393016D+00	0.419799D+00	0.448669D+00	0.479947D+00	0.514015D+00
0.62	0.386090D+00	0.412337D+00	0.440625D+00	0.471267D+00	0.504639D+00
0.63	0.379354D+00	0.405076D+00	0.432791D+00	0.462805D+00	0.495489D+00
0.64	0.372803D+00	0.398009D+00	0.425160D+00	0.454556D+00	0.486560D+00
0.65	0.366428D+00	0.391130D+00	0.417728D+00	0.446516D+00	0.477847D+00
0.66	0.360225D+00	0.384432D+00	0.410487D+00	0.438677D+00	0.469347D+00
0.67	0.354187D+00	0.377910D+00	0.403433D+00	0.431035D+00	0.461053D+00
0.68	0.348309D+00	0.371557D+00	0.396559D+00	0.423585D+00	0.452962D+00
0.69	0.342585D+00	0.365369D+00	0.389860D+00	0.416320D+00	0.445068D+00
0.70	0.337010D+00	0.359341D+00	0.383331D+00	0.409237D+00	0.437367D+00
0.71	0.331579D+00	0.353466D+00	0.376966D+00	0.402328D+00	0.429852D+00
0.72	0.326287D+00	0.347740D+00	0.370761D+00	0.395591D+00	0.422521D+00
0.73	0.321129D+00	0.342157D+00	0.364710D+00	0.389019D+00	0.415367D+00
0.74	0.316101D+00	0.336715D+00	0.358809D+00	0.382609D+00	0.408386D+00
0.75	0.311199D+00	0.331407D+00	0.353053D+00	0.376354D+00	0.401573D+00
0.76	0.306418D+00	0.326230D+00	0.347438D+00	0.370252D+00	0.394924D+00
0.77	0.301755D+00	0.321180D+00	0.341959D+00	0.364296D+00	0.388435D+00
0.78	0.297205D+00	0.316252D+00	0.336613D+00	0.358484D+00	0.382100D+00
0.79	0.292765D+00	0.311443D+00	0.331395D+00	0.352810D+00	0.375916D+00
0.80	0.288432D+00	0.306748D+00	0.326301D+00	0.347271D+00	0.369878D+00
0.81	0.284201D+00	0.302165D+00	0.321327D+00	0.341863D+00	0.363982D+00
0.82	0.280071D+00	0.297690D+00	0.316471D+00	0.336581D+00	0.358224D+00
0.83	0.276036D+00	0.293319D+00	0.311728D+00	0.331423D+00	0.352601D+00
0.84	0.272096D+00	0.289050D+00	0.307094D+00	0.326385D+00	0.347108D+00
0.85	0.268246D+00	0.284879D+00	0.302568D+00	0.321462D+00	0.341742D+00
0.86	0.264484D+00	0.280803D+00	0.298145D+00	0.316653D+0Ó	0.336500D+00
0.87	0.260807D+00	0.276820D+00	0.293823D+00	0.311953D+00	0.331377D+00
0.88	0.257212D+00	0.272926D+00	0.289598D+00	0.307359D+00	0.326370D+00
0.89	0.253698D+00	0.269119D+00	0.285467D+00	0.302869D+00	0.321477D+00
0.90	0.250261D+00	0.265397D+00	0.281429D+00	0.298479D+00	0.316693D+00
0.91	0.246900D+00	0.261756D+00	0.277480D+00	0.294187D+00	0.312017D+00
0.92	0.243612D+00	0.258195D+00	0.273617D+00	0.289989D+00	0.307444D+00
0.93	0.240395D+00	0.254711D+00	0.269838D+00	0.285883D+00	0.302972D+00
0.94	0.237246D+00	0.251302D+00	0.266142D+00	0.281867D+00	0.298598D+00
0.95	0.234165D+00	0.247966D+00	0.262524D+00	0.277937D+00	0.294320D+00
0.96	0.231148D+00	0.244701D+00	0.258984D+00	0.274092D+00	0.290135D+00
0.97	0.228195D+00	0.241504D+00	0.255519D+00	0.270329D+00	0.286040D+00
0.98	0.225303D+00	0.238374D+00	0.252127D+00	0.266646D+00	0.282032D+00
0.99	0.222470D+00	0.235309D+00	0.248805D+00	0.263040D+00	0.278110D+00
1.00	0.219696D+00	0.232307D+00	0.245553D+00	0.259510D+00	0.274271D+00
1.05	0.206637D+00	0.218184D+00	0.230259D+00	0.242923D+00	0.256245D+00
1.10	0.194806D+00	0.205400D+00	0.216430D+00	0.227940D+00	0.239984D+00
1.15	0.184050D+00	0.193788D+00	0.203881D+00	0.214362D+00	0.225269D+00
1.20	0.174239D+00	0.183206D+00	0.192458D+00	0.202019D+00	0.211912D+00
1.25	0.165261D+00	0.173533D+00	0.182029D+00	0.190764D+00	0.199752D+00
1.30	0.157022D+00	0.164666D+00	0.172480D+00	0.180473D+00	0.188651D+00
1.35	0.149441D+00	0.156514D+00	0.163712D+00	0.171037D+00	0.178488D+00
1.40	0.142446D+00	0.149002D+00	0.155642D+00	0.162363D+00	0.169161D+00
1.45	0.135977D+00	0.142061D+00	0.148195D+00	0.154371D+00	0.160580D+00
1.50	0.129980D+00	0.135634D+00	0.141307D+00	0.146989D+00	0.152667D+00
1.60	0.119222D+00	0.124123D+00	0.128993D+00	0.133818D+00	0.138579D+00
1.70	0.109864D+00	0.114131D+00	0.1Ì8329D+00	0.122442D+00	0.126447D+00
1.80	0.101664D+00	0.105393D+00	0.109026D+00	0.112542D+00	0.115921D+00
1.90	0.944323D−01	0.977029D−01	0.100855D+00	0.103869D+00	0.106724D+00
2.00	0.880163D−01	0.908931D−01	0.936357D−01	0.962246D−01	0.986376D−01
2.10	0.822933D−01	0.848301D−01	0.872213D−01	0.894477D−01	0.914877D−01
2.20	0.771631D−01	0.794049D−01	0.814931D−01	0.834090D−01	0.851319D−01
2.30	0.725433D−01	0.745282D−01	0.763538D−01	0.780025D−01	0.794545D−01
2.40	0.683658D−01	0.701257D−01	0.717229D−01	0.731406D−01	0.743602D−01
2.50	0.645737D−01	0.661358D−01	0.675334D−01	0.687506D−01	0.697701D−01
2.60	0.611190D−01	0.625067D−01	0.637292D−01	0.647718D−01	0.656182D−01
2.70	0.579611D−01	0.591944D−01	0.602630D−01	0.611529D−01	0.618492D−01
2.80	0.550658D−01	0.561619D−01	0.570945D−01	0.578505D−01	0.584163D−01
2.90	0.524033D−01	0.533774D−01	0.541895D−01	0.548278D−01	0.552798D−01
3.00	0.499484D−01	0.508134D−01	0.515186D−01	0.520532D−01	0.524055D−01
3.10	0.476792D−01	0.484464D−01	0.490565D−01	0.494993D−01	0.497643D−01
3.20	0.455765D−01	0.462561D−01	0.467812D−01	0.471427D−01	0.473309D−01
3.30	0.436238D−01	0.442244D−01	0.446736D−01	0.449629D−01	0.450837D−01
3.40	0.418064D−01	0.423360D−01	0.427171D−01	0.429422D−01	0.430035D−01
3.50	0.401118D−01	0.405770D−01	0.408971D−01	0.410650D−01	0.410737D−01
3.60	0.385285D−01	0.389355D−01	0.392007D−01	0.393175D−01	0.392799D−01
3.70	0.370466D−01	0.374009D−01	0.376165D−01	0.376877D−01	0.376090D−01
3.80	0.356572D−01	0.359636D−01	0.361345D−01	0.361650D−01	0.360499D−01
3.90	0.343525D−01	0.346153D−01	0.347459D−01	0.347397D−01	0.345925D−01
4.00	0.331254D−01	0.333485D−01	0.334426D−01	0.334037D−01	0.332279D−01
4.10	0.319697D−01	0.321565D−01	0.322175D−01	0.321492D−01	0.319482D−01
4.20	0.308795D−01	0.310333D−01	0.310643D−01	0.309695D−01	0.307462D−01
4.30	0.298499D−01	0.299734D−01	0.299773D−01	0.298587D−01	0.296155D−01
4.40	0.288762D−01	0.289720D−01	0.289512D−01	0.288114D−01	0.285506D−01
4.50	0.279543D−01	0.280248D−01	0.279815D−01	0.278225D−01	0.275462D−01
4.60	0.270804D−01	0.271276D−01	0.270639D−01	0.268876D−01	0.265977D−01
4.70	0.262511D−01	0.262770D−01	0.261947D−01	0.260029D−01	0.257009D−01
4.80	0.254632D−01	0.254695D−01	0.253703D−01	0.251646D−01	0.248519D−01
4.90	0.247140D−01	0.247022D−01	0.245876D−01	0.243694D−01	0.240474D−01
5.00	0.240008D−01	0.239724D−01	0.238438D−01	0.236144D−01	0.232842D−01

*t*	*β*				
	0.60	0.62	0.64	0.66	0.68
0.04	0.753752D−08				
0.05	0.378924D−05	0.731813D−08			
0.06	0.147234D−03	0.200982D−05	0.223739D−08		
0.07	0.153136D−02	0.690699D−04	0.553632D−06		
0.08	0.749966D−02	0.739405D−03	0.210256D−04	0.716141D−07	
0.09	0.230965D−01	0.390108D−02	0.261311D−03	0.370366D−05	0.337870D−08
0.10	0.525756D−01	0.130560D−01	0.160026D−02	0.609465D−04	0.304556D−06
0.11	0.974210D−01	0.321607D−01	0.612268D−02	0.474054D−03	0.786366D−05
0.12	0.156134D+00	0.639525D−01	0.169288D−01	0.221323D−02	0.878290D−04
0.13	0.225217D+00	0.109008D+00	0.371360D−01	0.721405D−02	0.548432D−03
0.14	0.300398D+00	0.165831D+00	0.687774D−01	0.181315D−01	0.226080D−02
0.15	0.377571D+00	0.231546D+00	0.112233D+00	0.375915D−01	0.687742D−02
0.16	0.453305D+00	0.302712D+00	0.166304D+00	0.673907D−01	0.166711D−01
0.17	0.525031D+00	0.375960D+00	0.228682D+00	0.108041D+00	0.339983D−01
0.18	0.591020D+00	0.448380D+00	0.296526D+00	0.158755D+00	0.606643D−01
0.19	0.650257D+00	0.517691D+00	0.366947D+00	0.217746D+00	0.974831D−01
0.20	0.702287D+00	0.582258D+00	0.437343D+00	0.282650D+00	0.144155D+00
0.21	0.747063D+00	0.641029D+00	0.505567D+00	0.350926D+00	0.199419D+00
0.22	0.784823D+00	0.693432D+00	0.569988D+00	0.420159D+00	0.261354D+00
0.23	0.815983D+00	0.739269D+00	0.629467D+00	0.488254D+00	0.327720D+00
0.24	0.841061D+00	0.778617D+00	0.683296D+00	0.553518D+00	0.396241D+00
0.25	0.860627D+00	0.811740D+00	0.731122D+00	0.614686D+00	0.464822D+00
0.26	0.875259D+00	0.839022D+00	0.772863D+00	0.670886D+00	0.531670D+00
0.27	0.885522D+00	0.860919D+00	0.808641D+00	0.721591D+00	0.595351D+00
0.28	0.891949D+00	0.877921D+00	0.838719D+00	0.766554D+00	0.654798D+00
0.29	0.895035D+00	0.890521D+00	0.863450D+00	0.805748D+00	0.709280D+00
0.30	0.895233D+00	0.899201D+00	0.883243D+00	0.839311D+00	0.758360D+00
0.31	0.892950D+00	0.904420D+00	0.898532D+00	0.867494D+00	0.801842D+00
0.32	0.888549D+00	0.906603D+00	0.909753D+00	0.890626D+00	0.839719D+00
0.33	0.882355D+00	0.906144D+00	0.917336D+00	0.909082D+00	0.872131D+00
0.34	0.874651D+00	0.903399D+00	0.921689D+00	0.923258D+00	0.899317D+00
0.35	0.865688D+00	0.898690D+00	0.923194D+00	0.933558D+00	0.921589D+00
0.36	0.855684D+00	0.892304D+00	0.922207D+00	0.940375D+00	0.939301D+00
0.37	0.844830D+00	0.884496D+00	0.919051D+00	0.944087D+00	0.952826D+00
0.38	0.833291D+00	0.875493D+00	0.914022D+00	0.945052D+00	0.962548D+00
0.39	0.821211D+00	0.865495D+00	0.907384D+00	0.943600D+00	0.968840D+00
0.40	0.808712D+00	0.854677D+00	0.899374D+00	0.940037D+00	0.972067D+00
0.41	0.795902D+00	0.843192D+00	0.890205D+00	0.934642D+00	0.972571D+00
0.42	0.782871D+00	0.831174D+00	0.880064D+00	0.927667D+00	0.970673D+00
0.43	0.769698D+00	0.818739D+00	0.869116D+00	0.919340D+00	0.966670D+00
0.44	0.756450D+00	0.805989D+00	0.857510D+00	0.909865D+00	0.960834D+00
0.45	0.743183D+00	0.793011D+00	0.845372D+00	0.899424D+00	0.953415D+00
0.46	0.729946D+00	0.779880D+00	0.832815D+00	0.888178D+00	0.944635D+00
0.47	0.716780D+00	0.766662D+00	0.819938D+00	0.876270D+00	0.934698D+00
0.48	0.703717D+00	0.753412D+00	0.806826D+00	0.863827D+00	0.923784D+00
0.49	0.690788D+00	0.740178D+00	0.793553D+00	0.850960D+00	0.912054D+00
0.50	0.678016D+00	0.727000D+00	0.780183D+00	0.837766D+00	0.899653D+00
0.51	0.665419D+00	0.713913D+00	0.766773D+00	0.824331D+00	0.886707D+00
0.52	0.653015D+00	0.700945D+00	0.753369D+00	0.810729D+00	0.873328D+00
0.53	0.640814D+00	0.688120D+00	0.740012D+00	0.797024D+00	0.859616D+00
0.54	0.628828D+00	0.675460D+00	0.726738D+00	0.783273D+00	0.845656D+00
0.55	0.617063D+00	0.662979D+00	0.713575D+00	0.769524D+00	0.831526D+00
0.56	0.605526D+00	0.650692D+00	0.700549D+00	0.755819D+00	0.817290D+00
0.57	0.594219D+00	0.638610D+00	0.687681D+00	0.742193D+00	0.803007D+00
0.58	0.583146D+00	0.626740D+00	0.674987D+00	0.728676D+00	0.788727D+00
0.59	0.572308D+00	0.615090D+00	0.662482D+00	0.715295D+00	0.774491D+00
0.60	0.561704D+00	0.603663D+00	0.650177D+00	0.702071D+00	0.760337D+00
0.61	0.551334D+00	0.592463D+00	0.638082D+00	0.689022D+00	0.746296D+00
0.62	0.541197D+00	0.581493D+00	0.626204D+00	0.676163D+00	0.732396D+00
0.63	0.531289D+00	0.570751D+00	0.614547D+00	0.663506D+00	0.718658D+00
0.64	0.521608D+00	0.560240D+00	0.603116D+00	0.651060D+00	0.705101D+00
0.65	0.512152D+00	0.549957D+00	0.591913D+00	0.638834D+00	0.691742D+00
0.66	0.502916D+00	0.539901D+00	0.580940D+00	0.626834D+00	0.678592D+00
0.67	0.493897D+00	0.530069D+00	0.570196D+00	0.615063D+00	0.665663D+00
0.68	0.485090D+00	0.520460D+00	0.559682D+00	0.603524D+00	0.652962D+00
0.69	0.476492D+00	0.511069D+00	0.549396D+00	0.592220D+00	0.640495D+00
0.70	0.468098D+00	0.501895D+00	0.539336D+00	0.581150D+00	0.628268D+00
0.71	0.459903D+00	0.492932D+00	0.529499D+00	0.570314D+00	0.616282D+00
0.72	0.451904D+00	0.484177D+00	0.519884D+00	0.559713D+00	0.604541D+00
0.73	0.444095D+00	0.475626D+00	0.510487D+00	0.549343D+00	0.593044D+00
0.74	0.436472D+00	0.467275D+00	0.501304D+00	0.539202D+00	0.581792D+00
0.75.	0.429030D+00	0.459120D+00	0.492333D+00	0.529289D+00	0.570783D+00
0.76	0.421766D+00	0.451156D+00	0.483568D+00	0.519600D+00	0.560017D+00
0.77	0.414674D+00	0.443379D+00	0.475006D+00	0.510131D+00	0.549490D+00
0.78	0.407749D+00	0.435784D+00	0.466643D+00	0.500880D+00	0.539200D+00
0.79	0.400989D+00	0.428368D+00	0.458475D+00	0.491841D+00	0.529145D+00
0.80	0.394387D+00	0.421125D+00	0.450497D+00	0.483012D+00	0.519319D+00
0.81	0.387941D+00	0.414052D+00	0.442705D+00	0.474387D+00	0.509721D+00
0.82	0.381645D+00	0.407145D+00	0.435095D+00	0.465964D+00	0.500345D+00
0.83	0.375497D+00	0.400398D+00	0.427663D+00	0.457737D+00	0.491189D+00
0.84	0.369491D+00	0.393809D+00	0.420403D+00	0.449702D+00	0.482246D+00
0.85	0.363624D+00	0.387372D+00	0.413313D+00	0.441854D+00	0.473513D+00
0.86	0.357892D+00	0.381084D+00	0.406387D+00	0.434191D+00	0.464986D+00
0.87	0.352292D+00	0.374941D+00	0.399623D+00	0.426706D+.00	0.456660D+00
0.88	0.346820D+00	0.368940D+00	0.393014D+00	0.419396D+00	0.448530D+00
0.89	0.341472D+00	0.363076D+00	0.386558D+00	0.412256D+00	0.440592D+00
0.90	0.336245D+00	0.357345D+00	0.380251D+00	0.405283D+00	0.432842D+00
0.91	0.331136D+00	0.351744D+00	0.374089D+00	0.398472D+00	0.425274D+00
0.92	0.326141D+00	0.346271D+00	0.368067D+00	0.391818D+00	0.417884D+00
0.93	0.321257D+00	0.340920D+00	0.362183D+00	0.385319D+00	0.410668D+00
0.94	0.316481D+00	0.335689D+00	0.356432D+00	0.378969D+00	0.403621D+00
0.95	0.311811D+00	0.330575D+00	0.350811D+00	0.372765D+00	0.396740D+00
0.96	0.307243D+00	0.325574D+00	0.345317D+00	0.366703D+00	0.390020D+00
0.97	0.302775D+00	0.320684D+00	0.339946D+00	0.360780D+00	0.383456D+00
0.98	0.298404D+00	0.315902D+00	0.334695D+00	0.354992D+00	0.377044D+00
0.99	0.294127D+00	0.311224D+00	0.329561D+00	0.349334D+00	0.370782D+00
1.00	0.289941D+00	0.306647D+00	0.324541D+00	0.343805D+00	0.364664D+00
1.05	0.270305D+00	0.285199D+00	0.301039D+00	0.317957D+00	0.336112D+00
1.10	0.252620D+00	0.265918D+00	0.279956D+00	0.294828D+00	0.310641D+00
1.15	0.236643D+00	0.248533D+00	0.260990D+00	0.274075D+00	0.287857D+00
1.20	0.222166D+00	0.232811D+00	0.243879D+00	0.255404D+00	0.267422D+00
1.25	0.209010D+00	0.218553D+00	0.228397D+00	0.238556D+00	0.249042D+00
1.30	0.197020D+00	0.205586D+00	0.214351D+00	0.223312D+00	0.232463D+00
1.35	0.186065D+00	0.193762D+00	0.201572D+00	0.209481D+00	0.217468D+00
1.40	0.176028D+00	0.182951D+00	0.189916D+00	0.196898D+00	0.203867D+00
1.45	0.166810D+00	0.173043D+00	0.179257D+00	0.185422D+00	0.191499D+00
1.50	0.158324D+00	0.163939D+00	0.169486D+00	0.174928D+00	0.180222D+00
1.60	0.143256D+00	0.147820D+00	0.152240D+00	0.156473D+00	0.160471D+00
1.70	0.130322D+00	0.134036D+00	0.137553D+00	0.140831D+00	0.143817D+00
1.80	0.119135D+00	0.122155D+00	0.124945D+00	0.127461D+00	0.129653D+00
1.90	0.109391D+00	0.111842D+00	0.114041D+00	0.115946D+00	0.117510D+00
2.00	0.100849D+00	0.102830D+00	0.104546D+00	0.105958D+00	0.107023D+00
2.10	0.933166D−01	0.949066D−01	0.962261D−01	0.972392D−01	0.979051D−01
2.20	0.866384D−01	0.879024D−01	0.888946D−01	0.895821D−01	0.899282D−01
2.30	0.806879D−01	0.816785D−01	0.823994D−01	0.828207D−01	0.829094D−01
2.40	0.753614D−01	0.761218D−01	0.766169D−01	0.768197D−01	0.767010D−01
2.50	0.705730D−01	0.711388D−01	0.714454D−01	0.714686D−01	0.711823D−01
2.60	0.662512D−01	0.666521D−01	0.668009D−01	0.666760D−01	0.662545D−01
2.70	0.623362D−01	0.625969D−01	0.626132D−01	0.623662D−01	0.618356D−01
2.80	0.587775D−01	0.589186D−01	0.588236D−01	0.584758D−01	0.578575D−01
2.90	0.555321D−01	0.555711D−01	0.553825D−01	0.549515D−01	0.542629D−01
3.00	0.525636D−01	0.525153D−01	0.522479D−01	0.517484D−01	0.510037D−01
3.10	0.49.8407D−01	0.497176D−01	0.493837D−01	0.488280D−01	0.480391D−01
3.20	0.473363D−01	0.471491D−01	0.467594D−01	0.461576D−01	0.453343D−01
3.30	0.450273D−01	0.447850D−01	0.443484D−01	0.437092D−01	0.428594D−01
3.40	0.428932D−01	0.426038D−01	0.421279D−01	0.414584D−01	0.405889D−01
3.50	0.409165D−01	0.405867D−01	0.400779D−01	0.393843D−01	0.385005D−01
3.60	0.390817D−01	0.387172D−01	0.381811D−01	0.374685D−01	0.365752D−01
3.70	0.373751D−01	0.369811D−01	0.364224D−01	0.356951D−01	0.347961D−01
3.80	0.357849D−01	0.353656D−01	0.347884D−01	0.340502D−01	0.331486D−01
3.90	0.343003D−01	0.338596D−01	0.332673D−01	0.325213D−01	0.316199D−01
4.00	0.329120D−01	0.324531D−01	0.318489D−01	0.310977D−01	0.301986D−01
4.10	0.316117D−01	0.311375D−01	0.305239D−01	0.297697D−01	0.288748D−01
4.20	0.303919D−01	0.299049D−01	0.292841D−01	0.285289D−01	0.276397D−01
4.30	0.292458D−01	0.287482D−01	0.281222D−01	0.273676D−01	00.264854D−01
4.40	0.281675D−01	0.276613D−01	0.270316D−01	0.262791D−01	0.254048D−01
4.50	0.271517D−01	0.266384D−01	0.260066D−01	0.252572D−01	0.243918D−01
4.60	0.261933D−01	0.256746D−01	0.250419D−01	0.242966D−01	0.234407D−01
4.70	0.252882D−01	0.247652D−01	0.241327D−01	0.233924D−01	0.225465D−01
4.80	0.244323D−01	0.239062D−01	0.232748D−01	0.225401D−01	0.217046D−01
4.90	0.236219D−01	0.230937D−01	0.224643D−01	0.217359D−01	0.209111D−01
5.00	0.228539D−01	0.223245D−01	0.216977D−01	0.209759D−01	0.201622D−01

*t*	*β*				
	0.70	0.72	0.74	0.76	0.78
0.11	0.802539D−08				
0.12	0.416131D−06				
0.13	0.801084D−05	0.535037D−08			
0.14	0.768242D−04	0.237760D−06			
0.15	0.445589D−03	0.437149D−05	0.107936D−08		
0.16	0.178343D−02	0.423297D−04	0.555972D−07		
0.17	0.540032D−02	0.254785D−03	0.120269D−05		
0.18	0.132108D−01	0.107115D−02	0.136544D−04	0.410099D−08	
0.19	0.273863D−01	0.342000D−02	0.952427D−04	0.132391D−06	
0.20	0.498423D−01	0.881655D−02	0.457233D−03	0.211103D−05	
0.21	0.817871D−01	0.192061D−01	0.164189D−02	0.196205D−04	0.353918D−08
0.22	0.123485D+00	0.365905D−01	0.469218D−02	0.120010D−03	0.103722D−06
0.23	0.174261D+00	0.625878D−01	0.111829D−01	0.528740D−03	0.158778D−05
0.24	0.232686D+00	0.980918D−01	0.230376D−01	0.179620D−02	0.146465D−04
0.25	0.296847D+00	0.143121D+00	0.421740D−01	0.495599D−02	0.906410D−04
0.26	0.364621D+00	0.196860D+00	0.701107D−01	0.115610D−01	0.408322D−03
0.27	0.433905D+00	0.257841D+00	0.107666D+00	0.235257D−01	0.142562D−02
0.28	0.502783D+00	0.324180D+00	0.154827D+00	0.428024D−01	0.405009D−02
0.29	0.569621D+00	0.393822D+00	0.210783D+00	0.710024D−01	0.972639D−02
0.30	0.633115D+00	0.464738D+00	0.274087D+00	0.109084D+00	0.203496D−01
0.31	0.692288D+00	0.535079D+00	0.342878D+00	0.157188D+00	0.379935D−01
0.32	0.746468D+00	0.603260D+00	0.415108D+00	0.214639D+00	0.645330D−01
0.33	0.795254D+00	0.668005D+00	0.488734D+00	0.280076D+00	0.101284D+00
0.34	0.838463D+00	0.728348D+00	0.561872D+00	0.351658D+00	0.148762D+00
0.35	0.876092D+00	0.783619D+00	0.632888D+00	0.427292D+00	0.206607D+00
0.36	0.908274D+00	0.833406D+00	0.700445D+00	0.504840D+00	0.273657D+00
0.37	0.935238D+00	0.877515D+00	0.763518D+00	0.582284D+00	0.348137D+00
0.38	0.957285D+00	0.915930D+00	0.821377D+00	0.657835D+00	0.427889D+00
0.39	0.974756D+00	0.948772D+00	0.873563D+00	0.730005D+00	0.510608D+00
0.40	0.988018D+00	0.976262D+00	0.919839D+00	0.797622D+00	0.594035D+00
0.41	0.997443D+00	0.998694D+00	0.960161D+00	0.859834D+00	0.676112D+00
0.42	0.100340D+01	0.101641D+01	0.994629D+00	0.916075D+00	0.755071D+00
0.43	0.100625D+01	0.102977D+01	0.102346D+01	0.966034D+00	0.829487D+00
0.44	0.100634D+01	0.103916D+01	0.104694D+01	0.100961D+01	0.898284D+00
0.45	0.100398D+01	0.104496D+01	0.106542D+01	0.104688D+01	0.960716D+00
0.46	0.999474D+00	0.104752D+01	0.107928D+01	0.107803D+01	0.101634D+01
0.47	0.993096D+00	0.104720D+01	0.108891D+01	0.110337D+01	0.106495D+01
0.48	0.985095D+00	0.104434D+01	0.109471D+01	0.112326D+01	0.110657D+01
0.49	0.975701D+00	0.103923D+01	0.109706D+01	0.113809D+01	0.114138D+01
0.50	0.965119D+00	0.103218D+01	0.109634D+01	0.114829D+01	0.116967D+01
0.51	0.953534D+00	0.102342D+01	0.109290D+01	0.115430D+01	0.119182D+01
0.52	0.941112D+00	0.101322D+01	0.108707D+01	0.115653D+01	0.120828D+01
0.53	0.927999D+00	0.100177D+01	0.107915D+01	0.115539D+01	0.121951D+01
0.54	0.914327D+00	0.989279D+00	0.106943D+01	0.115127D+01	0.122600D+01
0.55	0.900212D+00	0.975915D+00	0.105816D+01	0.114454D+01	0.122823D+01
0.56	0.885755D+00	0.961835D+00	0.104558D+01	0.113554D+01	0.122666D+01
0.57	0.871047D+00	0.947176D+00	0.103189D+01	0.112457D+01	0.122174D+01
0.58	0.856167D+00	0.932061D+00	0.101728D+01	0.111192D+01	0.121388D+01
0.59	0.841183D+00	0.916597D+00	0.100192D+01	0.109785D+01	0.120348D+01
0.60	0.826154D+00	0.900879D+00	0.985952D+00	0.108258D+01	0.119089D+01
0.61	0.811133D+00	0.884991D+00	0.969517D+00	0.106634D+01	0.117643D+01
0.62	0.796165D+00	0.869005D+00	0.952726D+00	0.104930D+01	0.116041D+01
0.63	0.781287D+00	0.852983D+00	0.935682D+00	0.103163D+01	0.114309D+01
0.64	0.766533D+00	0.836982D+00	0.918475D+00	0.101347D+01	0.112470D+01
0.65	0.751931D+00	0.821048D+00	0.901183D+00	0.994947D+00	0.110545D+01
0.66	0.737504D+00	0.805221D+00	0.883872D+00	0.976182D+00	0.108554D+01
0.67	0.723272D+00	0.789536D+00	0.866603D+00	0.957269D+00	0.106513D+01
0.68	0.709251D+00	0.774023D+00	0.849425D+00	0.938292D+00	0.104436D+01
0.69	0.695454D+00	0.758706D+00	0.832381D+00	0.919326D+00	0.102337D+01
0.70	0.681893D+00	0.743606D+00	0.815508D+00	0.900434D+00	0.100225D+01
0.71	0.668576D+00	0.728739D+00	0.798836D+00	0.881670D+00	0.981101D+00
0.72	0.655509D+00	0.714120D+00	0.782393D+00	0.863080D+00	0.960011D+00
0.73	0.642696D+00	0.699759D+00	0.766198D+00	0.844704D+00	0.939045D+00
0.74	0.630141D+00	0.685666D+00	0.750270D+00	0.826575D+00	0.918264D+00
0.75	0.617847D+00	0.671846D+00	0.734623D+00	0.808718D+00	0.897716D+00
0.76	0.605812D+00	0.658304D+00	0.719268D+00	0.791158D+00	0.877445D+00
0.77	0.594038D+00	0.645043D+00	0.704213D+00	0.773910D+00	0.857483D+00
0.78	0.582524D+00	0.632065D+00	0.689465D+00	0.756991D+00	0.837860D+00
0.79	0.571266D+00	0.619370D+00	0.675027D+00	0.740409D+00	0.818599D+00
0.80	0.560263D+00	0.606957D+00	0.660903D+00	0.724174D+00	0.799716D+00
0.81	0.549513D+00	0.594826D+00	0.647093D+00	0.708292D+00	0.781226D+00
0.82	0.539010D+00	0.582973D+00	0.633597D+00	0.692764D+00	0.763138D+00
0.83	0.528753D+00	0.571395D+00	0.620414D+00	0.677594D+00	0.745461D+00
0.84	0.518736D+00	0.560090D+00	0.607541D+00	0.662780D+00	0.728197D+00
0.85	0.508956D+00	0.549054D+00	0.594976D+00	0.648322D+00	0.711349D+00
0.86	0.499407D+00	0.538281D+00	0.582714D+00	0.634217D+00	0.694917D+00
0.87	0.490086D+00	0.527768D+00	0.570751D+00	0.620462D+00	0.678898D+00
0.88	0.480987D+00	0.517509D+00	0.559083D+00	0.607051D+00	0.663290D+00
0.89	0.472106D+00	0.507500D+00	0.547704D+00	0.593981D+00	0.648089D+00
0.90	0.463438D+00	0.497735D+00	0.536609D+00	0.581245D+00	0.633288D+00
0.91	0.454978D+00	0.488210D+00	0.525793D+00	0.568838D+00	0.618882D+00
0.92	0.446721D+00	0.478918D+00	0.515248D+00	0.556752D+00	0.604863D+00
0.93	0.438662D+00	0.469854D+00	0.504970D+00	0.544982D+00	0.591224D+00
0.94	0.430796D+00	0.461013D+00	0.494952D+00	0.533521D+00	0.577958D+00
0.95	0.423119D+00	0.452390D+00	0.485189D+00	0.522361D+00	0.565056D+00
0.96	0.415625D+00	0.443978D+00	0.475673D+00	0.511495D+00	0.552509D+00
0.97	0.408311D+00	0.435774D+00	0.466399D+00	0.500916D+00	0.540308D+00
0.98	0.401170D+00	0.427770D+00	0.457360D+00	0.490617D+00	0.528446D+00
0.99	0.394200D+00	0.419963D+00	0.448551D+00	0.480590D+00	0.516914D+00
1.00	0.387395D+00	0.412347D+00	0.439966D+00	0.470828D+00	0.505701D+00
1.05	0.355701D+00	0.376959D+00	0.400185D+00	0.425753D+00	0.454141D+00
1.10	0.327524D+00	0.345630D+00	0.365142D+00	0.386282D+00	0.409320D+00
1.15	0.302412D+00	0.317828D+00	0.334203D+00	0.351647D+00	0.370279D+00
1.20	0.279972D+00	0.293092D+00	0.306816D+00	0.321173D+00	0.336177D+00
1.25	0.259863D+00	0.271019D+00	0.282501D+00	0.294279D+00	0.306293D+00
1.30	0.241790D+00	0.251266D+00	0.260848D+00	0.270466D+00	0.280013D+00
1.35	0.225501D+00	0.233536D+00	0.241504D+00	0.249312D+00	0.256820D+00
1.40	0.210779D+00	0.217574D+00	0.224170D+00	0.230456D+00	0.236276D+00
1.45	0.197435D+00	0.203163D+00	0.208590D+00	0.213595D+00	0.218014D+00
1.50	0.185309D+00	0.190115D+00	0.194544D+00	0.198469D+00	0.201723D+00
1.60	0.164168D+00	0.167488D+00	0.170330D+00	0.172569D+00	0.174046D+00
1.70	0.146448D+00	0.148647D+00	0.150320D+00	0.151348D+00	0.151590D+00
1.80	0.131460D+00	0.132810D+00	0.133615D+00	0.133771D+00	0.133151D+00
1.90	0.118676D+00	0.119378D+00	0.119538D+00	0.119063D+00	0.117846D+00
2.00	0.107688D+00	0.107895D+00	0.107573D+00	0.106644D+00	0.105016D+00
2.10	0.981775D−01	0.980035D−01	0.973231D−01	0.960682D−01	0.941615D−01
2.20	0.898914D−01	0.894253D−01	0.884781D−01	0.869920D−01	0.849027D−01
2.30	0.826289D−01	0.819389D−01	0.807947D−01	0.791477D−01	0.769447D−01
2.40	0.762284D−01	0.753671D−01	0.740791D−01	0.723236D−01	0.700569D−01
2.50	0.705586D−01	0.695672D−01	0.681760D−01	0.663512D−01	0.640573D−01
2.60	0.655121D−01	0.644229D−01	0.629600D−01	0.610953D−01	0.588004D−01
2.70	0.610005D−01	0.598390D−01	0.583285D−01	0.564461D−01	0.541690D−01
2.80	0.569507D−01	0.557369D−01	0.541974D−01	0.523138D−01	0.500683D−01
2.90	0.533013D−01	0.520511D−01	0.504972D−01	0.486248D−01	0.464202D−01
3.00	0.500009D−01	0.487271D−01	0.471699D−01	0.453179D−01	0.431608D−01
3.10	0.470062D−01	0.457187D−01	0.441669D−01	0.423421D−01	0.402368D−01
3.20	0.442803D−01	0.429871D−01	0.414474D−01	0.396545D−01	0.376038D−01
3.30	0.417916D−01	0.404992D−01	0.389765D−01	0.372191D−01	0.352243D−01
3.40	0.395133D−01	0.382266D−01	0.367248D−01	0.350052D−01	0.330669D−01
3.50	0.374220D−01	0.361450D−01	0.346670D−01	0.329867D−01	0.311046D−01
3.60	0.354977D−01	0.342335D−01	0.327812D−01	0.311411D−01	0.293147D−01
3.70	0.337228D−01	0.324738D−01	0.310489D−01	0.294492D−01	0.276774D−01
3.80	0.320821D−01	0.308502D−01	0.294536D−01	0.278943D−01	0.261758D−01
3.90	0.305623D−01	0.293489D−01	0.279812D−01	0.264619D−01	0.247953D−01
4.00	0.291516D−01	0.279578D−01	0.266192D−01	0.251394D−01	0.235232D−01
4.10	0.278398D−01	0.266662D−01	0.253569D−01	0.239158D−01	0.223483D−01
4.20	0.266176D−01	0.254649D−01	0.241847D−01	0.227815D−01	0.212610D−01
4.30	0.254771D−01	0.243454D−01	0.230940D−01	0.217278D−01	0.202527D−01
4.40	0.244109D−01	0.233005D−01	0.220776D−01	0.207473D−01	0.193159D−01
4.50	0.234128D−01	0.223236D−01	0.211286D−01	0.198332D−01	0.184439D−01
4.60	0.224768D−01	0.214088D−01	0.202413D−01	0.189798D−01	0.176310D−01
4.70	0.215980D−01	0.205510D−01	0.194103D−01	0.181816D−01	0.168717D−01
4.80	0.207717D−01	0.197454D−01	0.186309D−01	0.174340D−01	0.161616D−01
4.90	0.199937D−01	0.189879D−01	0.178990D−01	0.167328D−01	0.154963D−01
5.00	0.192603D−01	0.182746D−01	0.172106D−01	0.160741D−01	0.148722D−01

*t*	*β*				
	0.80	0.82	0.84	0.86	0.88
0.25	0.255413D−07				
0.26	0.477184D−06				
0.27	0.525457D−05				
0.28	0.379878D−04	0.126195D−08			
0.29	0.195935D−03	0.393014D−07			
0.30	0.769172D−03	0.661087D−06			
0.31	0.241777D−02	0.679523D−05			
0.32	0.633452D−02	0.469762D−04			
0.33	0.142827D−01	0.235440D−03	0.134598D−07		
0.34	0.284347D−01	0.907667D−03	0.277183D−06		
0.35	0.510312D−01	0.282134D−02	0.338285D−05		
0.36	0.839632D−01	0.734202D−02	0.270323D−04		
0.37	0.128408D+00	0.164880D−01	0.153019D−03		
0.38	0.184615D+00	0.327469D−01	0.653185D−03	0.211304D−07	
0.39	0.251870D+00	0.586822D−01	0.221037D−02	0.429655D−06	
0.40	0.328623D+00	0.964459D−01	0.617305D−02	0.519765D−05	
0.41	0.412712D+00	0.147350D+00	0.146982D−01	0.412273D−04	
0.42	0.501631D+00	0.211615D+00	0.306364D−01	0.231645D−03	
0.43	0.592782D+00	0.288329D+00	0.571221D−01	0.980769D−03	0.391931D−08
0.44	0.683687D+00	0.375601D+00	0.969762D−01	0.328848D−02	0.123064D−06
0.45	0.772133D+00	0.470836D+00	0.152115D+00	0.908959D−02	0.209758D−05
0.46	0.856271D+00	0.571057D+00	0.223145D+00	0.213979D−01	0.217958D−04
0.47	0.934646D+00	0.673206D+00	0.309230D+00	0.440573D−01	0.151403D−03
0.48	0.100620D+01	0.774397D+00	0.408235D+00	0.810860D−01	0.756786D−03
0.49	0.107025D+01	0.872089D+00	0.517052D+00	0.135811D+00	0.288761D−02
0.50	0.112644D+01	0.964190D+00	0.632014D+00	0.210091D+00	0.882059D−02
0.51	0.117466D+01	0.104910D+01	0.749308D+00	0.303876D+00	0.224174D−01
0.52	0.121504D+01	0.112569D+01	0.865316D+00	0.415177D+00	0.489103D−01
0.53	0.124788D+01	0.119327D+01	0.976859D+00	0.540408D+00	0.939817D−01
0.54	0.127358D+01	0.125155D+01	0.108134D+01	0.674938D+00	0.162409D+00
0.55	0.129264D+01	0.130052D+01	0.117680D+01	0.813706D+00	0.256789D+00
0.56	0.130560D+01	0.134044D+01	0.126191D+01	0.951773D+00	0.376807D+00
0.57	0.131301D+01	0.137174D+01	0.133590D+01	0.108473D+01	0.519216D+00
0.58	0.131544D+01	0.139498D+01	0.139849D+01	0.120895D+01	0.678437D+00
0.59	0.131345D+01	0.141080D+01	0.144980D+01	0.132171D+01	0.847512D+00
0.60	0.130757D+01	0.141988D+01	0.149023D+01	0.142118D+01	0.101910D+01
0.61	0.129829D+01	0.142291D+01	0.152043D+01	0.150636D+01	0.118632D+01
0.62	0.128607D+01	0.142059D+01	0.154116D+01	0.157692D+01	0.134336D+01
0.63	0.127135D+01	0.141356D+01	0.155330D+01	0.163310D+01	0.148574D+01
0.64	0.125450D+01	0.140245D+01	0.155772D+01	0.167555D+01	0.161045D+01
0.65	0.123589D+01	0.138785D+01	0.155534D+01	0.170523D+01	0.171579D+01
0.66	0.121583D+01	0.137028D+01	0.154702D+01	0.172324D+01	0.180120D+01
0.67	0.119460D+01	0.135023D+01	0.153357D+01	0.173081D+01	0.186705D+01
0.68	0.117244D+01	0.132813D+01	0.151577D+01	0.172918D+01	0.191436D+01
0.69	0.114958D+01	0.130438D+01	0.149432D+01	0.171957D+01	0.194460D+01
0.70	0.112621D+01	0.127931D+01	0.146985D+01	0.170315D+01	0.195951D+01
0.71	0.110249D+01	0.125324D+01	0.144294D+01	0.168100D+01	0.196094D+01
0.72	0.107858D+01	0.122643D+01	0.141408D+01	0.165410D+01	0.195076D+01
0.73	0.105459D+01	0.119910D+01	0.138373D+01	0.162336D+01	0.193074D+01
0.74	0.103064D+01	0.117146D+01	0.135226D+01	0.158956D+01	0.190258D+01
0.75	0.100681D+01	0.114369D+01	0.132002D+01	0.155340D+01	0.186780D+01
0.76	0.983178D+00	0.111592D+01	0.128730D+01	0.151549D+01	0.182776D+01
0.77	0.959818D+00	0.108828D+01	0.125433D+01	0.147635D+01	0.178366D+01
0.78	0.936781D+00	0.106087D+01	0.122133D+01	0.143642D+01	0.173656D+01
0.79	0.914109D+00	0.103379D+01	0.118848D+01	0.139608D+01	0.168733D+01
0.80	0.891840D+00	0.100711D+01	0.115592D+01	0.135565D+01	0.163673D+01
0.81	0.870002D+00	0.980871D+00	0.112377D+01	0.131539D+01	0.158540D+01
0.82	0.848618D+00	0.955136D+00	0.109214D+01	0.127552D+01	0.153386D+01
0.83	0.827706D+00	0.929938D+00	0.106109D+01	0.123622D+01	0.148254D+01
0.84	0.807276D+00	0.905305D+00	0.103069D+01	0.119762D+01	0.143177D+01
0.85	0.787338D+00	0.881259D+00	0.100100D+01	0.115984D+01	0.138185D+01
0.86	0.767897D+00	0.857814D+00	0.972040D+00	0.112296D+01	0.133297D+01
0.87	0.748953D+00	0.834979D+00	0.943843D+00	0.108704D+01	0.128531D+01
0.88	0.730507D+00	0.812759D+00	0.916425D+00	0.105213D+01	0.123899D+01
0.89	0.712555D+00	0.791156D+00	0.889796D+00	0.101827D+01	0.119408D+01
0.90	0.695094D+00	0.770166D+00	0.863958D+00	0.985464D+00	0.115066D+01
0.91	0.678116D+00	0.749785D+00	0.838910D+00	0.953725D+00	0.110874D+01
0.92	0.661615D+00	0.730004D+00	0.814646D+00	0.923050D+00	0.106835D+01
0.93	0.645582D+00	0.710816D+00	0.791156D+00	0.893431D+00	0.102948D+01
0.94	0.630007D+00	0.692210D+00	0.768428D+00	0.864854D+00	0.992123D+00
0.95	0.614883D+00	0.674173D+00	0.746446D+00	0.837299D+00	0.956246D+00
0.96	0.600197D+00	0.656693D+00	0.725195D+00	0.810745D+00	0.921820D+00
0.97	0.585939D+00	0.639756D+00	0.704657D+00	0.785167D+00	0.888808D+00
0.98	0.572099D+00	0.623348D+00	0.684812D+00	0.760537D+00	0.857168D+00
0.99	0.558665D+00	0.607456D+00	0.665641D+00	0.736827D+00	0.826855D+00
1.00	0.545627D+00	0.592064D+00	0.647125D+00	0.714008D+00	0.797822D+00
1.05	0.485978D+00	0.522098D+00	0.563645D+00	0.612226D+00	0.670177D+00
1.10	0.434586D+00	0.462487D400	0.493521D+00	0.528288D+00	0.567472D+00
1.15	0.390223D+00	0.411597D+00	0.434485D+00	0.458877D+00	0.484524D+00
1.20	0.351812D+00	0.368008D+00	0.384593D+00	0.401205D+00	0.417110D+00
1.25	0.318437D+00	0.330522D+00	0.342228D+00	0.353007D+00	0.361904D+00
1.30	0.289325D+00	0.298143D+00	0.306070D+00	0.312473D+00	0.316332D+00
1.35	0.263830D+00	0.270049D+00	0.275044D+00	0.278165D+00	0.278410D+00
1.40	0.241413D+00	0.245560D+00	0.248281D+00	0.248941D+00	0.246604D+00
1.45	0.221623D+00	0.224118D+00	0.225074D+00	0.223894D+00	0.219726D+00
1.50	0.204086D+00	0.205261D+00	0.204848D+00	0.202298D+00	0.196849D+00
1.60	0.174558D+00	0.173841D+00	0.171553D+00	0.167250D+00	0.160341D+00
1.70	0.150864D+00	0.148949D+00	0.145564D+00	0.140356D+00	0.132883D+00
1.80	0.131604D+00	0.128942D+00	0.124942D+00	0.119331D+00	0.111778D+00
1.90	0.115758D+00	0.112648D+00	0.108338D+00	0.102618D+00	0.952449D−01
2.00	0.102582D+00	0.992200D−01	0.947904D−01	0.891342D−01	0.820738D−01
2.10	0.915160D−01	0.880336D−01	0.836055D−01	0.781120D−01	0.714248D−01
2.20	0.821391D−01	0.786235D−01	0.742718D−01	0.689949D−01	0.627009D−01
2.30	0.741283D−01	0.706373D−01	0.664074D−01	0.613730D−01	0.554699D−01
2.40	0.672332D−01	0.638046D−01	0.597227D−01	0.549400D−01	0.494130D−01
2.50	0.612577D−01	0.579156D−01	0.539953D−01	0.494633D−01	0.442915D−01
2.60	0.560464D−01	0.528057D−01	0.490523D−01	0.447638D−01	0.399238D−01
2.70	0.514752D−01	0.483441D−01	0.447578D−01	0.407023D−01	0.361699D−01
2.80	0.474440D−01	0.444264D−01	0.410039D−01	0.371691D−01	0.329207D−01
2.90	0.438712D−01	0.409680D−01	0.377040D−01	0.340768D−01	0.300901D−01
3.00	0.406902D−01	0.379002D−01	0.347881D−01	0.313555D−01	0.276096D−01
3.10	0.378459D−01	0.351665D−01	0.321990D−01	0.289483D−01	0.254239D−01
3.20	0.352924D−01	0.327201D−01	0.298900D−01	0.268088D−01	0.234883D−01
3.30	0.329915D−01	0.305224D−01	0.278220D−01	0.248990D−01	0.217661D−01
3.40	0.309109D−01	0.285407D−01	0.259628D−01	0.231870D−01	0.202273D−01
3.50	0.290233D−01	0.267477D−01	0.242852D−01	0.216467D−01	0.188466D−01
3.60	0.273057D−01	0.251201D−01	0.227663D−01	0.202558D−01	0.176032D−01
3.70	0.257382D−01	0.236383D−01	0.213868D−01	0.189956D−01	0.164795D−01
3.80	0.243037D−01	0.222852D−01	0.201301D−01	0.178503D−01	0.154607D−01
3.90	0.229876D−01	0.210465D−01	0.189820D−01	0.168063D−01	0.145340D−01
4.00	0.217771D−01	0.199095D−01	0.179303D−01	0.158520D−01	0.136888D−01
4.10	0.206613D−01	0.188633D−01	0.169647D−01	0.149774D−01	0.129157D−01
4.20	0.196305D−01	0.178987D−01	0.160758D−01	0.141739D−01	0.122067D−01
4.30	0.186762D−01	0.170071D−01	0.152558D−01	0.134340D−01	0.115550D−01
4.40	0.177910D−01	0.161816D−01	0.144977D−01	0.127510D−01	0.109545D−01
4.50	0.169684D−01	0.154156D−01	0.137954D−01	0.121194D−01	0.104000D−01
4.60	0.162026D−01	0.147035D−01	0.131436D−01	0.115341D−01	0.988695D−02
4.70	0.154884D−01	0.140404D−01	0.125376D−01	0.109906D−01	0.941126D−02
4.80	0.148213D−01	0.134219D−01	0.119730D−01	0.104851D−01	0.896941D−02
4.90	0.141972D−01	0.128441D−01	0.114463D−01	0.100140D−01	0.855824D−02
5.00	0.136125D−01	0.123034D−01	0.109540D−01	0.957441D−02	0.817499D−02

*t*	*β*				
	0.90	0.92	0.94	0.96	0.98
0.49	0.175529D−08				
0.50	0.820397D−07				
0.51	0.187578D−05				
0.52	0.241437D−04				
0.53	0.195463D−03				
0.54	0.108692D−02				
0.55	0.445324D−02				
0.56	0.142197D−01	0.139846D−08			
0.57	0.370207D−01	0.105583D−06			
0.58	0.814990D−01	0.332932D−05			
0.59	0.156250D+00	0.526634D−04			
0.60	0.267214D+00	0.481944D−03			
0.61	0.415676D+00	0.285267D−02			
0.62	0.597664D+00	0.119205D−01			
0.63	0.804762D+00	0.376784D−01			
0.64	0.102585D+01	0.951387D−01			
0.65	0.124906D+01	0.200409D+00	0.161725D−06		
0.66	0.146347D+01	0.364567D+00	0.879749D−05		
0.67	0.166018D+01	0.588766D+00	0.190728D−03		
0.68	0.183281D+01	0.863064D+00	0.204369D−02		
0.69	0.197757D+01	0.116905D+01	0.127375D−01		
0.70	0.209293D+01	0.148449D+01	0.522574D−01		
0.71	0.217915D+01	0.178792D+01	0.155091D+00		
0.72	0.223785D+01	0.206190D+01	0.357914D+00		
0.73	0.227150D+01	0.229453D+01	0.678961D+00		
0.74	0.228302D+01	0.247956D+01	0.110508D+01	0.644329D−07	
0.75	0.227556D+01	0.261551D+01	0.159526D+01	0.159988D−04	
0.76	0.225220D+01	0.270449D+01	0.209580D+01	0.793681D−03	
0.77	0.221587D+01	0.275095D+01	0.255668D+01	0.125896D−01	
0.78	0.216924D+01	0.276060D+01	0.294194D+01	0.887758D−01	
0.79	0.211465D+01	0.273958D+01	0.323279D+01	0.350948D+00	
0.80	0.205417D+01	0.269392D+01	0.342548D+01	0.916457D+00	
0.81	0.198953D+01	0.262917D+01	0.352689D+01	0.177633D+01	
0.82	0.192220D+01	0.255023D+01	0.354994D+01	0.277714D+01	
0.83	0.185338D+01	0.246128D+01	0.350992D+01	0.371726D+01	
0.84	0.178405D+01	0.236580D+01	0.342203D+01	0.444662D+01	
0.85	0.171500D+01	0.226658D+01	0.329993D+01	0.490323D+01	0.569651D−07
0.86	0.164683D+01	0.216587D+01	0.315514D+01	0.509740D+01	0.471182D−03
0.87	0.158002D+01	0.206538D+01	0.299689D+01	0.507860D+01	0.642527D−01
0.88	0.151493D+01	0.196642D+01	0.283231D+01	0.490757D+01	0.902888D+00
0.89	0.145181D+01	0.186995D+01	0.266669D+01	0.464012D+01	0.358054D+01
0.90	0.139086D+01	0.177666D+01	0.250383D+01	0.432050D+01	0.700029D+01
0.91	0.133218D+01	0.168701D+01	0.234632D+01	0.398056D+01	0.921090D+01
0.92	0.127584D+01	0.160128D+01	0.219584D+01	0.364151D+01	0.971591D+01
0.93	0.122186D+01	0.151963D+01	0.205339D+01	0.331644D+01	0.907158D+01
0.94	0.117025D+01	0.144210D+01	0.191947D+01	0.301261D+01	0.793277D+01
0.95	0.112096D+01	0.136868D+01	0.179422D+01	0.273340D+01	0.671051D+01
0.96	0.107396D+01	0.129928D+01	0.167752D+01	0.247970D+01	0.559410D+01
0.97	0.102917D+01	0.123378D+01	0.156910D+01	0.225091D+01	0.464461D+01
0.98	0.986518D+00	0.117203D+01	0.146856D+01	0.204556D+01	0.386369D+01
0.99	0.945935D+00	0.111387D+01	0.137548D+01	0.186177D+01	0.323073D+01
1.00	0.907332D+00	0.105912D+01	0.128936D+01	00.169753D+01	0.271997D+01
1.05	0.741019D+00	0.830273D+00	0.946793D+00	0.110334D+01	0.128811D+01
1.10	0.611719D+00	0.661154D+00	0.713482D+00	0.755736D+00	0.716959D+00
1.15	0.510612D+00	0.534903D+00	0.551345D+00	0.542306D+00	0.447586D+00
1.20	0.430831D+00	0.439309D+00	0.435858D+00	0.404585D+00	0.302938D+00
1.25	0.367219D+00	0.3658091+00	0.351547D+00	0.311677D+00	0.217435D+00
1.30	0.315955D+00	0.308434D+00	0.288562D+00	0.246554D+00	0.163107D+00
1.35	0.274204D+00	0.262995D+00	0.240516D+00	0.199394D+00	0.126609D+00
1.40	0.239857D+00	0.226524D+00	0.203171D+00	0.164281D+00	0.100986D+00
1.45	0.211332D+00	0.196886D+00	0.173652D+00	0.137506D+00	0.823451D−01
1.50	0.187431D+00	0.172528D+00	0.149968D+00	0.116666D+00	0.683815D−01
1.60	0.150048D+00	0.135340D+00	0.114859D+00	0.868623D−01	0.492615D−01
1.70	0.122586D+00	0.108773D+00	0.905940D−01	0.670597D−01	0.371293D−01
1.80	0.101890D+00	0.892001D−01	0.731814D−01	0.532722D−01	0.289650D−01
1.90	0.859419D−01	0.743997D−01	0.602917D−01	0.433063D−01	0.232152D−01
2.00	0.734156D−01	0.629564D−01	0.504986D−01	0.358786D−01	0.190167D−01
2.10	0.634102D−01	0.539376D−01	0.428925D−01	0.301997D−01	0.158591D−01
2.20	0.552997D−01	0.467104D−01	0.368724D−01	0.257635D−01	0.134256D−01
2.30	0.486391D−01	0.408340D−01	0.320290D−01	0.222338D−01	0.115110D−01
2.40	0.431054D−01	0.359941D−01	0.280763D−01	0.193803D−01	0.997794D−02
2.50	0.384601D−01	0.319622D−01	0.248098D−01	0.170415D−01	0.873155D−02
2.60	0.345241D−01	0.285691D−01	0.220801D−01	0.151009D−01	0.770466D−02
2.70	0.311609D−01	0.256874D−01	0.197761D−01	0.134733D−01	0.684867D−02
2.80	0.282652D−01	0.232197D−01	0.178142D−01	0.120950D−01	0.612771D−02
2.90	0.257547D−01	0.210907D−01	0.161300D−01	0.109177D−01	0.551483D−02
3.00	0.235642D−01	0.192415D−01	0.146736D−01	0.990411D−02	0.498948D−02
3.10	0.216417D−01	0.176251D−01	0.134059D−01	0.902539D−02	0.453577D−02
3.20	0.199455D−01	0.162043D−01	0.122956D−01	0.825862D−02	0.414124D−02
3.30	0.184415D−01	0.149487D−01	0.113178D−01	0.758561D−02	0.379605D−02
3.40	0.171017D−01	0.138338D−01	0.104523D−01	0.699167D−02	0.349229D−02
3.50	0.159032D−01	0.128394D−01	0.968251D−02	0.646491D−02	0.322361D−02
3.60	0.148268D−01	0.119487D−01	0.899487D−02	0.599557D−02	0.298479D−02
3.70	0.138565D−01	0.111478D−01	0.837809D−02	0.557560D−02	0.277158D−02
3.80	0.129788D−01	0.104250D−01	0.782277D−02	0.519832D−02	0.258044D−02
3.90	0.121823D−01	0.977054D−02	0.732102D−02	0.485814D−02	0.240842D−02
4.00	0.114572D−01	0.917606D−02	0.686616D−02	0.455034D−02	0.225307D−02
4.20	0.101896D−01	0.813959D−02	0.607530D−02	0.401658D−02	0.198432D−02
4.40	0.912234D−02	0.727000D−02	0.541399D−02	0.357168D−02	0.176098D−02
4.60	0.821535D−02	0.653328D−02	0.485541D−02	0.319696D−02	0.157337D−02
4.80	0.743806D−02	0.590366D−02	0.437933D−02	0.287840D−02	0.141425D−02
5.00	0.676682D−02	0.536134D−02	0.397025D−02	0.260530D−02	0.127813D−02

*t*	*β*				
	0.990	0.995	0.997	0.998	0.999
0.9250	0.304435D−01				
0.9300	0.724474D+00				
0.9350	0.413781D+01				
0.9400	0.103257D+02				
0.9450	0.159373D+02				
0.9460	0.167331D+02				
0.9470	0.173985D+02				
0.9480	0.179335D+02				
0.9490	0.183414D+02				
0.9500	0.186282D+02				
0.9510	0.188019D+02				
0.9520	0.188719D+02				
0.9530	0.188481D+02				
0.9540	0.187412D+02				
0.9550	0.185613D+02				
0.9560	0.183187D+02				
0.9570	0.180228D+02				
0.9580	0.176827D+02	0.207732D−01			
0.9590	0.173064D+02	0.109476D+00			
0.9600	0.169014D+02	0.413897D+00			
0.9610	0.164744D+02	0.119543D+01			
0.9620	0.160311D+02	0.277546D+01			
0.9630	0.155769D+02	0.539843D+01			
0.9640	0.151160D+02	0.909628D+01			
0.9650	0.146524D+02	0.136434D+02			
0.966G	0.141892D+02	0.186215D+02			
0.9670	0.137293D+02	0.235457D+02			
0.9680	0.132750D+02	0.279852D+02			
0.9690	0.128279D+02	0.316365D+02			
0.9700	0.123898D+02	0.343449D+02			
0.9710	0.119618D+02	0.360858D+02			
0.9720	0.115447D+02	0.369291D+02			
0.9730	0.111393D+02	0.370001D+02	0.224193D−01		
0.9740	0.107461D+02	0.364475D+02	0.327681D+00		
0.9750	0.103653D+02	0.354200D+02	0.209692D+01		
0.9760	0.999722D+01	0.340534D+02	0.747066D+01		
0.9770	0.964186D+01	0.324632D+02	0.175707D+02		
0.9780	0.929917D+01	0.307431D+02	0.307895D+02		
0.9790	0.896903D+01	0.289656D+02	0.438034D+02		
0.9800	0.865126D+01	0.271853D+02	0.537808D+02		
0.9805	0.849694D+01	0.263068D+02	0.571903D+02		
0.9810	0.834562D+01	0.254412D+02	0.595157D+02		
0.9815	0.819727D+01	0.245914D+02	0.608249D+02	0.160180D+00	
0.9820	0.805183D+01	0.237599D+02	0.612297D+02	0.889475D+00	
0.9825	0.790929D+01	0.229486D+02	0.608644D+02	0.326711D+01	
0.9830	0.776958D+01	0.221588D+02	0.598693D+02	0.871315D+01	
0.9835	0.763267D+01	0.213917D+02	0.583798D+02	0.181354D+02	
0.9840	0.749853D+01	0.206479D+02	0.565191D+02	0.311551D+02	
0.9845	0.736709D+01	0.199279D+02	0.543950D+02	0.461344D+02	
0.9850	0.723832D+01	0.192318D+02	0.520981D+02	0.609020D+02	
0.9855	0.711217D+01	0.185598D+02	0.497029D+02	0.735681D+02	
0.9860	0.698860D+01	0.179116D+02	0.472686D+02	0.829855D+02	
0.9865	0.686755D+01	0.172869D+02	0.448414D+02	0.887989D+02	
0.9870	0.674899D+01	0.166854D+02	0.424560D+02	0.912470D+02	
0.9875	0.663286D+01	0.161067D+02	0.401379D+02	0.909006D+02	
0.9880	0.651912D+01	0.155500D+02	0.379049D+02	0.884433D+02	
0.9885	0.640773D+01	0.150149D+02	0.357690D+02	0.845305D+02	
0.9890	0.629863D+01	0.145007D+02	0.337370D+02	0.797185D+02	
0.9895	0.619178D+01	0.140068D+02	0.318125D+02	0.744417D+02	0.128057D−02
0.9900	0.608714D+01	0.135325D+02	0.299960D+02	0.690183D+02	0.253893D+00
0.9901	0.606647D+01	0.134399D+02	0.296456D+02	0.679369D+02	0.542909D+00
0.9902	0.604589D+01	0.133480D+02	0.292994D+02	0.668599D+02	0.107406D+01
0.9903	0.602539D+01	0.132570D+02	0.289575D+02	0.657887D+02	0.198062D+01
0.9904	0.600498D+01	0.131666D+02	0.286198D+02	0.647244D+02	0.342758D+01
0.9905	0.598466D+01	0.130770D+02	0.282862D+02	0.636679D+02	0.560072D+01
0.9906	0.596442D+01	0.129881D+02	0.279569D+02	0.626202D+02	0.868902D+01
0.9907	0.594426D+01	0.128999D+02	0.276316D+02	0.615821D+02	0.128629D+02
0.9908	0.592419D+01	0.128125D+02	0.273105D+02	0.605543D+02	0.182520D+02
0.9909	0.590420D+01	0.127258D+02	0.269934D+02	0.595376D+02	0.249263D+02
0.9910	0.588429D+01	0.126397D+02	0.266804D+02	0.585324D+02	0.328840D+02
0.9911	0.586447D+01	0.125544D+02	0.263713D+02	0.575394D+02	0.420476D+02
0.9912	0.584473D+01	0.124698D+02	0.260663D+02	0.565589D+02	0.522680D+02
0.9913	0.582507D+01	0.123858D+02	0.257652D+02	0.555914D+02	0.633368D+02
0.9914	0.580549D+01	0.123026D+02	0.254679D+02	0.546371D+02	0.750017D+02
0.9915	0.578600D+01	0.122200D+02	0.251746D+02	0.536965D+02	0.869862D+02
0.9916	0.576658D+01	0.121381D+02	0.248850D+02	0.527696D+02	0.990079D+02
0.9917	0.574725D+01	0.120569D+02	0.245993D+02	0.518567D+02	0.110795D+03
0.9918	0.572799D+01	0.119763D+02	0.243173D+02	0.509580D+02	0.122101D+03
0.9919	0.570882D+01	0.118964D+02	0.240390D+02	0.500735D+02	0.132714D+03
0.9920	0.568973D+01	0.118171D+02	0.237644D+02	0.492033D+02	0.142460D+03
0.9921	0.567071D+01	0.117385D+02	0.234934D+02	0.483476D+02	0.151211D+03
0.9922	0.565178D+01	0.116605D+02	0.232260D+02	0.475062D+02	0.158879D+03
0.9923	0.563292D+01	0.115832D+02	0.229621D+02	0.466793D+02	0.165415D+03
0.9924	0.561415D+01	0.115065D+02	0.227018D+02	0.458667D+02	0.170805D+03
0.9925	0.559545D+01	0.114304D+02	0.224449D+02	0.450684D+02	0.175065D+03
0.9926	0.557682D+01	0.113549D+02	0.221914D+02	0.442844D+02	0.178235D+03
0.9927	0.555828D+01	0.112801D+02	0.219413D+02	0.435146D+02	0.180374D+03
0.9928	0.553981D+01	0.112058D+02	0.216946D+02	0.427588D+02	0.181552D+03
0.9929	0.552142D+01	0.111322D+02	0.214512D+02	0.420169D+02	0.181851D+03
0.9930	0.550311D+01	0.110592D+02	0.212110D+02	0.412888D+02	0.181356D+03
0.9931	0.548487D+01	0.109867D+02	0.209741D+02	0.405744D+02	0.180155D+03
0.9932	0.546671D+01	0.109149D+02	0.207403D+02	0.398734D+02	0.178335D+03
0.9933	0.544862D+01	0.108436D+02	0.205097D+02	0.391858D+02	0.175979D+03
0.9934	0.543061D+01	0.107729D+02	0.202821D+02	0.385114D+02	0.173166D+03
0.9935	0.541267D+01	0.107028D+02	0.200577D+02	0.378500D+02	0.169971D+03
0.9936	0.539481D+01	0.106333D+02	0.198362D+02	0.372013D+02	0.166463D+03
0.9937	0.537702D+01	0.105643D+02	0.196177D+02	0.365653D+02	0.162703D+03
0.9938	0.535930D+01	0.104959D+02	0.194022D+02	0.359417D+02	0.158747D+03
0.9939	0.534166D+01	0.104281D+02	0.191895D+02	0.353303D+02	0.154646D+03
0.9940	0.532409D+01	0.103608D+02	0.189798D+02	0.347310D+02	0.150444D+03
0.9942	0.528917D+01	0.102278D+02	0.185686D+02	0.335676D+02	0.141883D+03
0.9944	0.525454D+01	0.100970D+02	0.181685D+02	0.324499D+02	0.133313D+03
0.9946	0.522019D+01	0.996825D+01	0.177790D+02	0.313762D+02	0.124914D+03
0.9948	0.518613D+01	0.984161D+01	0.173999D+02	0.303450D+02	0.116809D+03
0.9950	0.515235D+01	0.971701D+01	0.170309D+02	0.293546D+02	0.109080D+03
0.9960	0.498757D+01	0.912334D+01	0.153274D+02	0.249607D+02	0.770202D+02
0.9970	0.482945D+01	0.857550D+01	0.138362D+02	0.213653D+02	0.550465D+02
0.9980	0.467770D+01	0.806958D+01	0.125283D+02	0.184123D+02	0.403125D+02
0.9990	0.453203D+01	0.760198D+01	0.113782D+02	0.159741D+02	0.303186D+02
1.0000	0.439217D+01	0.716940D+01	0.103644D+02	0.139490D+02	0.233847D+02
1.0010	0.425787D+01	0.676883D+01	0.946812D+01	0.122560D+02	0.184504D+02
1.0020	0.412887D+01	0.639755D+01	0.867348D+01	0.108317D+02	0.148511D+02
1.0030	0.400493D+01	0.605304D+01	0.796691D+01	0.962565D+01	0.121648D+02
1.0040	0.388584D+01	0.573305D+01	0.733683D+01	0.859805D+01	0.101177D+02
1.0050	0.377138D+01	0.543552D+01	0.677335D+01	0.771724D+01	0.852845D+01
1.0100	0.326150D+01	0.422493D+01	0.469747D+01	0.478245D+01	0.422422D+01
1.0200	0.248913D+01	0.271963D+01	0.258091D+01	0.229363D+01	0.162193D+01
1.0300	0.194690D+01	0.187297D+01	0.160578D+01	0.132261D+01	0.841468D+00
1.0400	0.155592D+01	0.135820D+01	0.108674D+01	0.853819D+00	0.511577D+00
1.0500	0.126694D+01	0.102519D+01	0.780848D+00	0.594368D+00	0.342832D+00
1.1000	0.557631D+00	0.365425D+00	0.247198D+00	0.175463D+00	0.935803D−01
1.2000	0.192128D+00	0.109266D+00	0.691426D−01	0.473530D−01	0.243272D−01
1.3000	0.949409D−01	0.513358D−01	0.317892D−01	0.215305D−01	0.109368D−01
1.4000	0.561814D−01	0.296348D−01	0.181638D−01	0.122385D−01	0.618439D−02
1.5000	0.370172D−01	0.192470D−01	0.117281D−01	0.787899D−02	0.396971D−02
1.6000	0.261897D−01	0.134914D−01	0.819031D−02	0.549201D−02	0.276190D−02
1.7000	0.194902D−01	0.997588D−02	0.604054D−02	0.404529D−02	0.203175D−02
1.8000	0.150625D−01	0.767343D−02	0.463770D−02	0.310293D−02	0.155700D−02
1.9000	0.119859D−01	0.608435D−02	0.367208D−02	0.245513D−02	0.123108D−02
2.0000	0.976264D−02	0.494191D−02	0.297928D−02	0.199084D−02	0.997720D−03
2.2000	0.683487D−02	0.344570D−02	0.207391D−02	0.138473D−02	0.693413D−03
2.4000	0.504998D−02	0.253861D−02	0.152623D−02	0.101848D−02	0.509728D−03
2.6000	0.388252D−02	0.194762D−02	0.116996D−02	0.780411D−03	0.390419D−03
2.8000	0.307752D−02	0.154129D−02	0.925282D−03	0.617008D−03	0.308575D−03
3.0000	0.249916D−02	0.125002D−02	0.750041D−03	0.500026D−03	0.250008D−03
3.2000	0.206974D−02	0.103414D−02	0.620249D−03	0.413413D−03	0.206660D−03
3.4000	0.174220D−02	0.869712D−03	0.521449D−03	0.347500D−03	0.173682D−03
3.6000	0.148669D−02	0.741601D−03	0.444507D−03	0.296181D−03	0.148011D−03
3.8000	0.128354D−02	0.639848D−03	0.383419D−03	0.255445D−03	0.127637D−03
4.0000	0.111937D−02	0.557690D−03	0.334112D−03	0.222570D−03	0.111199D−03
4.2000	0.984811D−03	0.490399D−03	0.293739D−03	0.195657D−03	0.977428D−04
4.4000	0.873143D−03	0.434595D−03	0.260267D−03	0.173346D−03	0.865894D−04
4.6000	0.779455D−03	0.387803D−03	0.232207D−03	0.154645D−03	0.772418D−04
4.8000	0.700083D−03	0.348183D−03	0.208453D−03	0.138815D−03	0.693300D−04
5.0000	0.632254D−03	0.314341D−03	0.188167D−03	0.125297D−03	0.625745D−04

**Table 3:** *t*_max_ and *g*(*t*_max_) as a function of *β*, where *g*(*t*) is the inverse Laplace transform of $e^{-s\beta }$

*β*	*t*_max_	*g*(*t*_max_)
0.15	0.00000190503	450.9129
0.16	0.00000630739	209.22449
0.17	0.0000177337	108.862395
0.18	0.0000436172	62.162073
0.19	0.0000960414	38.311543
0.20	0.000192795	25.155645
0.21	0.000357987	17.416529
0.22	0.000622088	12.610041
0.23	0.00102137	9.484013
0.24	0.00159686	7.369062
0.25	0.00239285	5.888686
0.26	0.00345532	4.821523
0.27	0.00483018	4.032270
0.28	0.00656175	3.435337
0.29	0.00869134	2.974907
0.30	0.0112561	2.613581
0.31	0.0142884	2.325679
0.32	0.0178147	2.093179
0.33	0.0218560	1.903181
0.34	0.0264274	1.746284
0.35	0.0315381	1.615524
0.36	0.0371920	1.505665
0.37	0.0433880	1.412716
0.38	0.0501201	1.333598
0.39	0.0573788	1.265907
0.40	0.0651506	1.207748
0.41	0.0734193	1.157611
0.42	0.0821662	1.114283
0.43	0.0913707	1.076782
0.44	0.101011	1.044308
0.45	0.111063	1.016202
0.46	0.121503	0.991922
0.47	0.132308	0.971016
0.48	0.143453	0.953108
0.49	0.154914	0.937885
0.50	0.166667	0.925082
0.51	0.178689	0.914477
0.52	0.190959	0.905885
0.53	0.203454	0.899150
0.54	0.216155	0.894139
0.55	0.229042	0.890746
0.56	0.242097	0.888881
0.57	0.255304	0.888472
0.58	0.268646	0.889461
0.59	0.282109	0.891806
0.60	0.295680	0.895475
0.61	0.309346	0.900451
0.62	0.323098	0.906726
0.63	0.336924	0.914305
0.64	0.350818	0.923202
0.65	0.364772	0.933445
0.66	0.378780	0.945070
0.67	0.392837	0.958129
0.68	0.406941	0.972686
0.69	0.421088	0.988819
0.70	0.435279	1.006623
0.71	0.449512	1.026212
0.72	0.463791	1.047719
0.73	0.478118	1.071303
0.74	0.492496	1.097151
0.75	0.506933	1.125483
0.76	0.521435	1.156560
0.77	0.536011	1.190689
0.78	0.550671	1.228239
0.79	0.565430	1.269648
0.80	0.580300	1.315445
0.81	0.595300	1.366273
0.82	0.610450	1.422918
0.83	0.625773	1.486349
0.84	0.641295	1.557781
0.85	0.657048	1.638750
0.86	0.673068	1.731228
0.87	0.689397	1.837792
0.88	0.706087	1.961869
0.89	0.723197	2.108126
0.90	0.740799	2.283081
0.91	0.758982	2.496129
0.92	0.777856	2.761327
0.93	0.797561	3.100708
0.94	0.818279	3.550899
0.95	0.840258	4.177648
0.96	0.863851	5.112078
0.97	0.889596	6.659241
0.98	0.918411	9.731437
0.990	0.9522311	18.874343
0.995	0.9725934	37.055102
0.997	0.9819976	61.229747
0.998	0.9871752	91.409296
0.999	0.9928860	181.858807
1.000	1.0000000	INFINITY
1/3	0.0233204	1.847590
2/3	0.388146	0.953613
0.56787475	0.25248543	0.888440006391162 **=lowest value of *g*(*t*_max_)**
